# Reduced engagement with social stimuli in 6-month-old infants with later autism spectrum disorder: a longitudinal prospective study of infants at high familial risk

**DOI:** 10.1186/s11689-016-9139-8

**Published:** 2016-03-15

**Authors:** E. J. H. Jones, K. Venema, R. Earl, R. Lowy, K. Barnes, A. Estes, G. Dawson, S. J. Webb

**Affiliations:** Centre for Brain and Cognitive Development, Birkbeck College, University of London, London, UK; Center on Human Development and Disability, University of Washington, Seattle, WA USA; Center for Child Health, Behavior and Development, Seattle Children’s Hospital, Seattle, WA USA; Department of Speech and Hearing Sciences, University of Washington, Seattle, WA USA; Department of Psychiatry and Behavioral Sciences, Duke University, Durham, NC USA; Department of Psychiatry and Behavioral Sciences, University of Washington, Seattle, WA USA

**Keywords:** ASD, Habituation, Event-related potential, Social attention, Social information processing

## Abstract

**Background:**

Autism spectrum disorder (ASD) is a neurodevelopmental disorder that affects more than 1 % of the population and close to 20 % of prospectively studied infants with an older sibling with ASD. Although significant progress has been made in characterizing the emergence of behavioral symptoms of ASD, far less is known about the underlying disruptions to early learning. Recent models suggest that core aspects of the causal path to ASD may only be apparent in early infancy. Here, we investigated social attention in 6- and 12-month-old infants who did and did not meet criteria for ASD at 24 months using both cognitive and electrophysiological methods. We hypothesized that a reduction in attention engagement to faces would be associated with later ASD.

**Methods:**

In a prospective longitudinal design, we used measures of both visual attention (habituation) and brain function (event-related potentials to faces and objects) at 6 and 12 months and investigated the relationship to ASD outcome at 24 months.

**Results:**

High-risk infants who met criteria for ASD at 24 months showed shorter epochs of visual attention, faster but less prolonged neural activation to faces, and delayed sensitization responses (increases in looking) to faces at 6 months; these differences were less apparent at 12 months. These findings are consistent with disrupted engagement of sustained attention to social stimuli.

**Conclusions:**

These findings suggest that there may be fundamental early disruptions to attention engagement that may have cascading consequences for later social functioning.

**Electronic supplementary material:**

The online version of this article (doi:10.1186/s11689-016-9139-8) contains supplementary material, which is available to authorized users.

## Background

Autism spectrum disorder (ASD) is a neurodevelopmental disorder that affects more than 1 % of the US population [1]. Individuals with ASD experience difficulty with social communication and display restrictive interests and repetitive behaviors [2, 3]. Reliable diagnosis of ASD can be made by 18 months to 3 years for most individuals, and the average age of diagnosis is around age 4 years in the USA [4], but parent concerns begin earlier, particularly if there is an older sibling with ASD in the family [5]. Understanding the causal paths to ASD requires studying infants prior to the onset of autism-specific behavioral symptoms [6]. Over the last 10 years, a number of investigators have begun to address these questions using prospective studies of infants with older siblings with ASD. Since infants with an older sibling with ASD have close to a 20 % risk of developing ASD themselves [7], researchers can examine the neural and cognitive precursors to symptom emergence by following a cohort of “infant siblings” from early infancy to early childhood.

Infant sibling studies have led to significant progress in characterizing the emergence of behavioral symptoms of ASD [6], in part replicating findings from earlier case report, parent report, and retrospective videotape studies [8–10]. Such work has broadly revealed that infants start to fall behind their peers in their social and communication skills early in the second half of the first year of life [11]. Findings hold significant promise for improvements in early screening [12]. However, far less is known about the disruptions to perception, attention, or learning that precede the progressive failure to develop social and communication skills at the typical pace in infants with later ASD. Shifting the level of analysis from behavioral symptoms to the developmental mechanisms that underlie their emergence is critical to the design of more effective interventions and will help to bridge the gap between genetics and clinical presentation.

Social attention models of ASD propose that deficits in social attention and orienting begin to emerge in the second half of the first year of life, leading to reduced engagement with social stimuli, and thus reduced opportunities for social learning [13–16]. These early deficits may thus have cascading effects on social communication development. Such models suggest that early social attention may be a fruitful target for early intervention. Thus, testing social attention/motivation models has been a strong focus of work with infant siblings [6]. The majority of such studies have focused on examining *where* infants direct their attention during naturalistic live and video-based social experiences because this reveals the type of information infants are sampling from their environment. Such work presents a mixed picture of early social attention in ASD. Some studies have observed disruptions in early social attention: for example, 6-month-old infants with later ASD show reduced visual attention to inner facial features when faces are speaking [17] and reduced attention to an actress in a naturalistic scene [18]. However, in other studies, 7- and 14-month-old infants show typical patterns of orienting to faces in static displays [19] and typical modulation of attention to different types of facial movement in complex social displays [20]. Other studies have observed gradual reductions in attention to the eyes of a naturalistic “caregiver” video between 2 and 24 months [21] and to faces during a live observational assessment between 6 and 36 months [11]. Reasons for the disparity in findings on the *direction* of attention over the first year of life remain unclear.

Developmental decreases in allocation of attention to social stimuli in ASD could be a consequence of earlier-emerging difficulties with processing social information [6, 14, 22]. Under such models, initial subcortically mediated social orienting mechanisms are intact in ASD [23], but difficulties with processing incoming social information make social experiences progressively less rewarding, leading to decreases in social attention over developmental time. Chawarksa and colleagues have argued that the depth of processing afforded to social stimuli may be atypical in infants with later ASD, causing cascading consequences for subsequent learning [24]. For example, they propose that while typically developing toddlers may examine a novel face and spontaneously compute its category (face or non-face?), familiarity (mother or stranger?), and affect (happy or sad?), toddlers with ASD may engage in more limited processing. This is expected to lead to poorer face learning because work with adults indicates that deeper processing facilitates later retention (e.g., [25]). In the hypothesis of reduced depth of processing for face stimuli, toddlers with ASD show more rapid disengagement from a face than an object stimulus [24], are less distracted by the presence of a face in a gaze cuing task [26], and show slowed face learning [22]. Further, toddlers with ASD show developmental delays in how facial familiarity modulates attention-related neural responses, and the extent of the developmental delay relates to their general social level [27]. However, to establish whether these disruptions could contribute to the emergence of ASD (rather than representing a consequence of spending less time attending to other people), it is necessary to examine whether they are present prior to ASD symptom expression. Thus, in the present study, we set out to test whether a reduced depth of attention to social stimuli is present in infants at high risk for ASD in the first year of life.

We selected two widely used paradigms to test this proposal. First, we used a habituation paradigm to examine the duration of individual epochs of attention to social and nonsocial stimuli. In a habituation task, infants are presented with a stimulus that is repeated until the infant’s looking declines to a predefined level. In such paradigms, the duration of the longest look to the stimulus produced prior to the habituation criteria partly reflects individual differences in sustained attention [28], with a longer peak look associated with higher levels of attention engagement to the stimulus. In typical development, individual differences in peak look duration are relatively reliable, show robust relations to long-term cognitive outcomes [29], and are stable across different screen-based paradigms [30]. Measurement of concurrent heart rate indicate that over 50 % of the duration of the infant’s peak look is spent in a state of “sustained attention” to the stimulus, and this proportion is particularly high around the age of 6 months [28]. A second related measure of attention engagement derived from habituation paradigms is the position of the peak look in the looking sequence. About two thirds of “typical” infants do not show a monotonic decrease in look duration during habituation [31]. The “dual-process” account [32] of habituation posits that in addition to progressive habituation to stimulus characteristics, an additional process of “sensitization” operates that is associated with a spike in parasympathetic arousal that *increases* attention to the stimulus [33]. Sensitization is thought to be important in engaging deeper levels of processing in response to communicative cues, including the facilitation of learning by infant-directed speech [34, 35]. Thus, a peak look that is later in the habituation sequence would indicate delayed sensitization to the stimulus. Taken together, a peak look that was shorter in duration and later in the habituation sequence would be associated with reduced attention engagement to social stimuli.

Secondly, we examined event-related potentials (ERPs) to faces and objects. In an ERP paradigm, EEG is continuously recorded while infants view briefly presented stimuli. The neural response time-locked to each stimulus presentation is averaged within each category, producing a characteristic pattern of components that are sensitive to the time-course of information processing. Such paradigms have already shown sensitivity to detecting atypical social processing in infants with later ASD; for example, 8-month-old infants with later ASD show an attenuated P400 response to shifts in gaze direction [36]. Here, we were interested in two components (the P400 and the Nc) that have been previously shown to be sensitive to depth of attention engagement and processing of social stimuli in ASD. The Nc is a negative-going deflection that peaks around 500 ms after the onset of a particular stimulus [37]. Because it is modulated by novelty [38], and stimulus salience [37], and is larger to stimuli presented during physiologically defined states of attention [39], the Nc is thought to reflect attention engagement [40]. Previous work with toddlers with ASD has shown that the modulation of the Nc by facial familiarity is atypical [27]. In the present study, we examined Nc amplitude (as a measure of initial depth of engagement) and the duration of the Nc as a measure of the degree to which attention was sustained. We predicted that a smaller and less sustained Nc component would reflect reduced attention engagement with faces in infants with later ASD.

Secondly, the P400 is a positive-going deflection that typically peaks around 300 to 600 ms after stimulus onset [40–42]. In infancy, this component is sensitive to complex aspects of face processing. For example, in typical development, the P400 is modulated by face inversion [43], dynamic gaze shifts [36], and peaks earlier and with smaller amplitude to faces than objects, consistent with greater attention capture or depth of processing by unfamiliar objects than faces in this age range [41, 44]. Taken together, researchers have argued that the P400 reflects the processing of semantic and structural aspects of faces and may be the precursor to the adult N170 [40]. We predicted that if infants with later ASD show reduced depth of engagement with faces, the P400 response to faces would peak even more rapidly and be of even smaller amplitude in infants with later ASD than in typically developing infants. Of note, a faster P400 latency to faces versus objects would replicate findings in a previous study of 6- to 10-month-old infants with later ASD [36].

We tested infants at 6 and 12 months because this represents the timescale over which clear symptoms of ASD in social and communication domains begin to emerge [6]. Thus, we were particularly interested in differences in attention engagement that may be apparent at 6 months and could thus potentially contribute to autism-specific symptom emergence. Interestingly, recent studies of high-risk infants have suggested that some deficits in basic aspects of development may be apparent in early infancy but appear to resolve in later development. For example, Libertus and colleagues [45] recently showed deficits in reaching and grasping in 6-month-old infants at high risk for ASD that apparently resolved at 10 months. Further, a recent large study also found motor delays at 6 months in infants with a later ASD diagnosis; these delays appeared to resolve by 12 months but emerged again by 24 months [46]. At older ages, deficits in more complex aspects of development may become more apparent (e.g., the onset of walking [47]). This may reflect transient delays in the acquisition of newly emerging skills that accumulate into cascading effects over the infancy period [6, 48]. Because our paradigms are simple and suitable for very young infants, it may similarly be that deficits would be detected at 6 months (representing a transient delay) but apparently resolved by 12 months. Of note, other previous studies that have observed deficits in social processing at 6 months [17, 18, 36] have not examined the same variables at older ages, making this an important question.

Although there has been a long tradition of work with typically developing infants using our paradigms, we first sought to establish that the particular test format we had chosen elicited the expected pattern of normative performance in a large group of typically developing infants at 6 and 12 months (Experiment 1). Comparison of our findings to previous work indicates that our paradigms produce the expected developmental effects. Secondly, in Experiment 2, we examined performance in an independent sample of infants at high and low familial risk from a prospective longitudinal study who did and did not later develop ASD.

## Experiment 1: Normative data

### Methods

#### Participants

Participants were 114 (51 females) 6-month-old and 104 12-month-old (50 females) typically developing full-term infants. Parents and their infants were recruited using the University of Washington Communication Studies Infant Participant Pool. Exclusionary criteria included a known family history of ASD in first- or second-degree relatives; physical signs (e.g., dysmorphic features) of known genetic syndromes; serious medical or neurological conditions (e.g., encephalitis, concussion, seizure disorder, diabetes, congenital heart disease); neurocutaneous markings or sensory impairments such as vision or hearing loss; serious motor impairment; birth weight <2000 g and/or gestational age <37 weeks; history of intraventricular hemorrhage, exposure to neurotoxins (including alcohol, drugs); and maternal gestational diabetes. In addition, variables that may impact family functioning (e.g., serious parental substance abuse, bipolar disorder, or psychosis) were exclusion criteria.

All infants participated in the event-related potential paradigm; approximately half the infants (51 6-month-olds, 27 females; 55 12-month-olds, 27 females) participated in the habituation paradigm. This approach was chosen because the expected attrition rate for EEG paradigms is approximately 50 % in this age range, and we sought approximately equivalent group sizes for the two analyses.

#### Habituation task

##### Habituation stimuli

Stimuli were colored photographs of female faces and objects, measuring 25 cm by 25 cm. Objects were chosen to be symmetrical, forward facing, and did not have any features that could be interpreted as representing a face. Four pairs of stimuli were used in each stimulus condition (faces or objects) to ensure findings were not item specific. Stimuli were counterbalanced across participants. Preliminary analysis confirmed group effects did not differ as a function of stimulus set, and so analyses were collapsed across this variable.

##### Habituation procedure

At 6 and 12 months, children participated in four habituation experiments, in a two-stimuli (faces or objects)-by-two-delay (1 s versus 1 min) repeated-measures design. Two delays were used to assess whether infants might show difficulties with immediate versus longer-term face or object recognition. Testing was conducted across two different days to reduce possible transfer effects. At each visit, one test involving faces and one test involving objects was presented. At 6 months, one test at each visit was conducted with the short delay (1 s), and one was conducted with the long delay (1 min) (for example: day 1 face long delay and object short delay; day 2 object long delay and face short delay.) At 12 months, both tasks at each visit were conducted with one delay (either long or short). Order of testing for both stimulus and delay was counterbalanced within these restrictions. Parents were asked to refrain from providing verbal or nonverbal cues during the testing procedure. Preliminary analysis confirmed that group effects did not significantly differ as a function of testing day or order, and so analyses presented were collapsed across these variables.

Infants were seated on their parent’s lap approximately 100 cm from the display; stimuli subtended 14° by 14° of visual angle and were presented on a 46-in. liquid crystal display monitor. A closed-circuit camera was placed underneath the monitor. Two experimenters stood behind a barrier and monitored the infant’s behavior via live feed from the camera.

Stimulus presentation was controlled using a custom-built software package (“LookTime”). During the habituation phase, two experimenters independently measured looking time by pressing a button while the child visually fixated the stimulus. The stimulus was removed from the screen if the child looked away for more than 1 s (based on online computation of data from the first experimenter). When this occurred, an “attention getter” (a flashing colored square accompanied by a chirping noise) was used to regain the child’s attention to the screen. When the child attended to the screen for longer than 1 s, the stimulus was represented. We used a reorient cue to maximize participant retention and to reduce the potential effect of differences in endogenous orienting on look spacing and habituation times [49]; for examples, see [50–53]. Reorienting cues are included in a leading program used to implement habituation protocols [54].

**Fig. 1:**
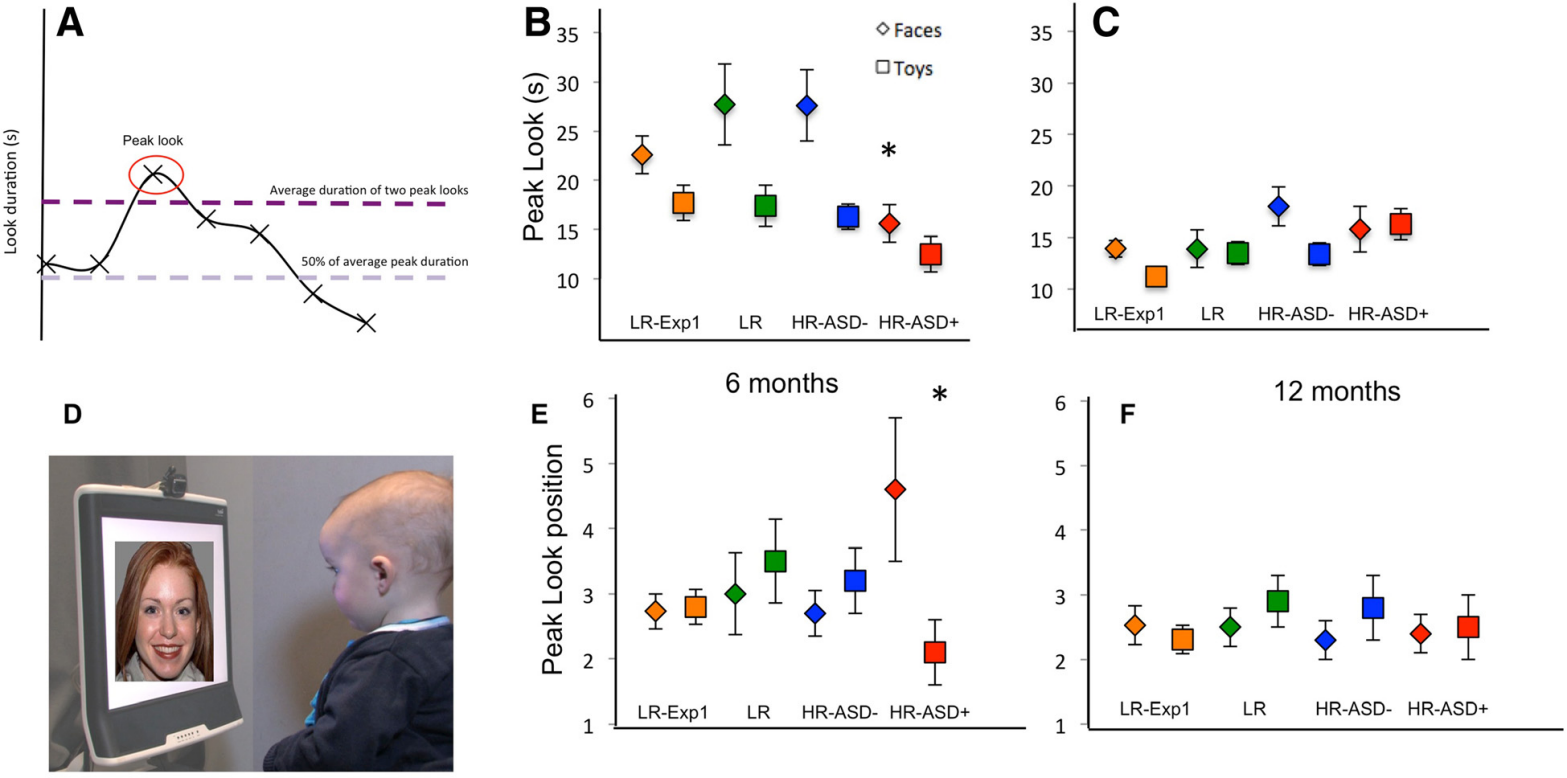
Habituation task at 6 and 12 months. **a** Illustration of an idealized habituation function, showing the peak look measure. **b** Peak look duration at 6 months in the four groups (low-risk control, HR-ASD+, and HR-ASD-Neg). **c** Peak look duration at 12 months. **d** Illustration of the habituation procedure. **e** Mean position of the peak look in the looking sequence (in **a**, the peak look is the third in the sequence) at 6 months. **f** Mean position of the peak look at 12 months. All error bars are ±1 standard error

Habituation was defined as having been met when each of two consecutive looks fell below 50 % of the average of the child’s longest two looks, requiring a minimum of four looks (illustrated in Fig. 1a). A “look” was defined as visual fixation for greater than 1 s. These calculations were implemented by the LookTime software. The two longest looks (rather than first look duration) were chosen as the criterion because around 40 % of individual infants produce their peak looks later in the habituation function [29, 31, 55]. We did not implement a cutoff time within which the infant had to reach the habituation criterion because we did not want to affect our sensitivity to individual differences; however, if infants became excessively fussy during testing, the experiment was terminated and marked as invalid.

The habituation phase was followed by a delay phase of either 1 s or 1 min. During the delay phase, the child was not shown any stimulus. In the testing phase, the familiar stimulus and a previously unseen stimulus of the same stimulus category were presented in random order. Each stimulus was presented for the duration of one look.

Habituation data was considered valid if (1) infants met the habituation criterion, (2) habituation was not judged to be invalid during testing (e.g., child was crying, or eyes could not be seen), and (3) look coding was considered reliable as assessed by calculating the intra-class correlation coefficient between the first and second experimenter coding for all infants. If the correlation was less than *r* = 0.8, the video recording was re-coded offline by trained coders. Additional file 1: Table S2 gives details of the number of valid habituation periods in each condition obtained from each group and indicates the number of habituations judged invalid, and Additional file 2: Text S1.4.1. gives additional information on participant validity. Additional file 1: Table S4 provides the average intra-class correlation coefficient for valid habituation sessions, illustrating the high level of agreement between the two coders.

##### Habituation data processing

As children participated in two face habituation experiments and two object habituation experiments at each time-point, summary values (e.g., peak look duration) were averaged across the two experiments in each condition to provide a more stable characterization of individual differences [56–58]. If a child had only one valid data point for either the object or the face condition, this data point alone was included in the analysis. This enabled us to maximize the number of children included in the final analysis. Preliminary tests revealed no significant effects of the number of data points included in the analysis for each child (all *p*s >0.1); this variable was excluded from further analyses.

We analyzed two key variables from the habituation phase of the experiment (Fig. 1a): duration of peak look during habituation and the position of the peak look in the sequence. These two aspects of the habituation function represent different processes, with look duration variables (e.g., peak look) representing influences of processing speed and sustained attention and peak look position representing the speed of “sensitization” [33].

##### Dishabituation

In order to establish that infants had indeed habituated to the stimulus presented (rather than general features of the test setting), the duration of looking to the novel stimulus was compared to the duration of the last look during habituation with a repeated-measures analysis. If the last look was significantly shorter than the look to the novel stimulus, dishabituation to the specific stimulus features was inferred.

##### Habituation analysis strategy

Valid data was obtained from 98 % of 6-month-old infants and 93 % of 12-month-old infants (see Additional file 1: Table S2 for full details of inclusion rates). Analyses of habituation variables (peak look duration, peak look position) included age (6 or 12 months) and gender (male, female) as between-subjects factors and stimulus (face or object) as the within-subject factor. Where significant interactions were found, follow-up univariate ANOVAs or paired *t* tests were used to clarify the pattern of findings. For dishabituation, we first used repeated-measures ANOVAs on looking times to the familiar versus novel stimulus for each age (6, 12 months), stimulus (face, object), and delay (short, long) condition separately. A significant effect of familiar versus novel indicates dishabituation in that paradigm. Second, we examined whether dishabituation magnitudes differed as a function of age, gender, stimulus, or delay in a repeated-measures ANOVA on looking times by familiarity (familiar, novel), age (6, 12 months), gender (male, female), stimulus (face, object), and delay (short, long).

#### Event-related potential task

##### Stimuli

**Fig. 2:**
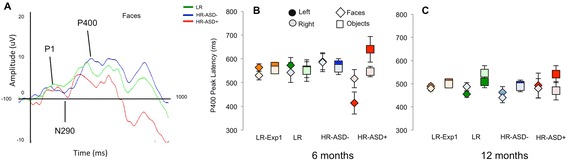
Posterior event-related potentials. **a** Illustration of the grand average event-related potential over left occipital electrodes at 6 months, showing the P1, N290, and P400 components. **b** Mean latency of the P400 response to faces or objects at 6 months in the four groups (low-risk control, HR-ASD+, and HR-ASD-Neg). **c** Mean latency of the P400 response to faces or objects at 12 months. All error bars are ±1 standard error

One hundred digital photographs of faces (including both internal and external features) and objects (objects) were presented. Face stimuli were chosen to reflect the ethnicity of the local community (86 % Caucasian, 8 % Asian, and 6 % African-American); gender was balanced. Objects were photographs of age-appropriate toddlers’ “favorite” objects that did not have a face and were oriented vertically to match faces in size and width [59], as depicted in Fig. 2a. Stimulus frames were 336 pixels wide by 420 pixels high and were presented for 500 ms on an LCD monitor 65 cm from the child at a size of 18 cm by 11 cm, subtending a visual angle of 16° by 10°.

##### ERP procedure

ERPs were recorded from 128-channel geodesic sensor nets recorded with reference to the vertex. Data was recorded at 500 Hz, with amplification set at ×1000, and band-pass filtering at 0.1 and 100 Hz. Children were presented with a series of 2300- to 2800-ms trials consisting of 100 ms baseline, 500 ms stimulus presentation, and 1200 ms post-stimulus recording period; 500–1000 ms randomly jittered ITI. Testing was terminated when the child had attended to 100 of each of the stimulus types or when the child was no longer attending. Offline, data were low-pass filtered at 20 Hz and segmented into 1800 ms epochs. Artifact detection was accomplished with both automatic artifact-detection software (NetStation 4.3) and through hand-editing. During hand-editing, files were labeled by subject number. Trials were rejected if the child did not attend to the picture (recorded online by a trained observer), if the signal amplitude exceeded 250 μV, if electro-ocular or muscular artifact occurred, or if there was a significant drift. Data was re-referenced offline to the average reference, and trials were corrected with respect to the 100 ms pre-stimulus baseline period.

Posterior temporal left and right regions for the P400 and the fronto-central region for the Nc (Additional file 1: Figure S1) and components of interest were defined with respect to the previous literature, and inspection of the grand average waveform. These regions substantially overlap those used in previous work with children with ASD [36, 41, 59–61]. For the P400, we analyzed peak amplitude and latency because these measures have been sensitive to atypicalities in infants with later ASD [36] and children with ASD [61]. Peaks were identified for each electrode using automatic peak detection software and verified by visual inspection. Peaks were defined as the most positive point of a deflection between 200 and 900 msec (P400), and the peak had to be present in at least two sixths electrodes in a group [36, 41, 44]. Peak amplitude and latency values were averaged across regions.

For the Nc, we analyzed two measures that have shown atypicalities in previous work with toddlers with ASD [27]. First, we examined mean amplitude as a measure of magnitude of attention engagement, with time windows selected based on the grand average of the normative data (Experiment 1) and previous work [27, 62]. Because the early and late sections of the Nc may reflect different neural sources [62], we separately examined amplitude within the early (300 to 600) and late (600 to 900) portions of the Nc component. Further, we examined the duration of attention engagement by examining the latency at which the ERP waveform (averaged over a 50-ms window for greater stability) crossed from negative to positive (i.e., the timing of the end of the Nc component).

##### ERP analysis strategy

Valid data was obtained from 44 % of 6-month-old infants and 58 % of 12-month-old infants (see Additional file 1: Table S3 for full details of inclusion rates). Components were initially analyzed in a series of repeated-measures ANOVAs, with age (6 or 12 months) and gender (male, female) as between-subject variables and within-subject variables of laterality (left, right) and stimulus (face, object). Greenhouse-Geisser corrections were used. Where significant interactions were found, follow-up univariate ANOVAs or paired *t* tests were used to clarify the pattern of findings.

##### Behavioral measures

To confirm that they were typically developing, 50 % of the infants (*n* = 50 6-month-olds and *n* = 54 12-month-olds) participated in the Mullen Scales of Early Learning (Mullen), a standardized developmental assessment that provides standard scores in the domains of visual reception, fine and gross motor skills, and receptive and expressive language. Data from these measures from all infants tested is presented in Additional file 1: Table S1 and indicates that as a group, the sample performed within the typical range.

## Results

### Habituation to faces and objects

#### Peak look duration

In a repeated-measures ANOVA on peak look duration by age (6 or 12 months), gender (male, female), and stimulus (face, object), peak look duration was longer to face than objects (*F*(1,100) = 9.40. *p* = 0.003, *η*^2^ = 0.09), and peak look duration was shorter at 12 months than 6 months (*F*(1,100) = 21.77, *p* < 0.001, *η*^2^ = 0.18). There was no significant interaction between stimulus and age (*F*(1,100) = 0.82, *p* = 0.37, *η*^2^ = 0.008), and no main effects or interactions with gender (*F*s < 1, *p*s > 0.3). These patterns are consistent with previous work [28, 29, 63], confirming that our paradigm was robustly designed.

#### Peak look position

In a repeated-measures ANOVA on peak look position by age (6 or 12 months), gender (male, female), and stimulus (face, object), there were no differences in the position of the peak look in the sequence for faces and objects (*F*(1,100) = 0.061, *p* = 0.81, *η*^2^ = 0.001) or for the two age groups (*F*(1,100) = 1.33. *p* = 0.25, *η*^2^ = 0.013) and no interaction with age and stimulus (*F*(1,100) = 0.33, *p* = 0.6, *η*^2^ = 0.003). However, broadly in line with previous work [50], 60 % of 6-month-olds and 50 % of 12-month-olds tended to produce their peak look after the first look in the habituation function. This confirms that our stimuli produce effects consistent with sensitization in a substantial proportion of infants.

#### Dishabituation

Finally, analysis of dishabituation times separately for each age group (6 and 12 months), delay (short, long), and stimulus (face, object) indicated that dishabituation magnitudes were significant for all tasks (*F*(1,68) = 126.5, *p* < 0.001). Repeated-measures ANOVAs on dishabituation magnitudes by age (6, 12 months), gender (male, female), stimulus (face, object), and delay interval (short, long) showed no significant interactions between familiarity and stimulus, delay interval, or age groups, indicating no general differences in dishabituation magnitude as a function of these factors (*F*s < 1, *p*s > 0.5). This confirms that habituation measures resulted from habituation to the specific stimulus presented, rather than general features of the test setting.

### P400 neural responses to faces and objects

In a repeated-measures ANOVA on P400 latency by age (6 or 12 months), gender (male, female), stimulus (face, object), and laterality (left, right), P400 latency peaked earlier to faces than objects (*F*(1,99) = 6.70, *p* = 0.011, *η*^2^ = 0.011), was faster over right than left electrodes (*F*(1,99) = 4.77, *p* = 0.031, *η*^2^ = 0.046), and had a shorter latency at 12 than 6 months (*F*(1,99) = 18.1, *p* < 0.001, *η*^2^ = 0.15). Male infants also showed faster P400 latencies than female infants (*M* female = 539.5, *M* male = 506.1; *F*(1,101) = 4.9, *p* = 0.03, *η*^2^ = 0.045). In a repeated-measures ANOVA on P400 amplitude by age (6 or 12 months), gender (male, female), stimulus (face, object), and laterality (left, right), P400 amplitude was greater to objects than faces (*F***(**1,99) = 23.4, *p* < 0.001, *η*^2^ = 0.19). This is consistent with previous work [41, 43, 64].

### Nc neural responses to faces and objects

In repeated-measures ANOVAs on Nc amplitude for early and late subcomponents separately by age (6 or 12 months), gender (male, female), and stimulus (face, object), Nc overall amplitude was more negative to objects than faces for both the early and late subcomponents (early *F*(1,104) = 9.9, *p* < 0.001, *η*^2^ = 0.09; late *F*(1,104) = 7.0, *p* = 0.009, *η*^2^ = 0.063). For the early Nc component, there was a significant interaction between age and gender (*F*(1,104) = 5.1, *p* = 0.026, *η*^2^ = 0.047) and a main effect of gender (early: *F*(1,104) = 4.01, *p* = 0.048, *η*^2^ = 0.037), driven by the fact that age-related change was significant in males (*F*(1,53) = 3.94, *p* = 0.05, *η*^2^ = 0.07) but not females (*F*(1,51) = 1.53, *p* = 0.22, *η*^2^ = 0.03). For the late Nc component, amplitudes were generally more negative at 12 than 6 months (*F*(1,104) = 10.3, *p* = 0.002, *η*^2^ = 0.09).

In repeated-measures ANOVAs on Nc duration by age (6 or 12 months), gender (male, female), and stimulus (face, object), the duration of the Nc was longer to objects than faces (*F*(1,84) = 3.9, *p* = 0.05, *η*^2^ = 0.045) and longer at 6 months than 12 months (*F*(1,84) = 19.9, *p* < 0.001, *η*^2^ = 0.19). Again, these results are comparable to previous work; for example, typically developing 3- to 4-year-old children show a more negative Nc to objects than faces [41].

### Correlations within neural responses

To establish which of the ERP findings were interrelated, we examined patterns of correlations between P400 latency and amplitude and Nc latency and amplitude to faces. At 6 months but not 12 months, faster P400 latency to faces over the left hemisphere was correlated with a shorter duration Nc to faces (*r*(41) = 0.38, *p* = 0.014) and a less negative Nc response to faces (*r*(50) = −0.4, *p* = 0.006). Similarly, a less negative Nc to faces was highly correlated with a shorter Nc latency (*r*(42) = −0.9, *p* < 0.001). Taken together, these findings confirm (as expected) that a fast P400 latency to faces and a shorter and less negative Nc are interrelated and may be associated with lesser attention capture by social stimuli.

### Summary

Experiment 1 confirmed that our paradigms elicit normative patterns of responding in typically developing infants. This includes a faster and smaller P400 to faces than objects, a smaller and shorter Nc to faces than objects, and a longer peak look to faces than objects during habituation.

In Experiment 2, we used these paradigms to examine differences in attention capture by faces and objects in infants at high risk for ASD. We reasoned that if attention capture by social stimuli were reduced in infants with later ASD, we would see an exaggeration of the faster P400 to faces and the smaller and shorter Nc to faces versus objects (reflecting an exaggeration of the typical tendency for greater attention capture by objects versus faces in this age range). Further, we predicted that we would see a reduction in the duration of the peak look to faces during the habituation paradigm.

## Experiment 2: infants at risk for autism

### Methods

#### Participants

Participants were recruited from the NIH-funded Early Connections project examining the development of infants at high or low risk for ASD. All procedures were carried out in accordance with ethical approval granted by the local Institutional Review Board. High-risk (HR) infants had an older sibling with a clinical diagnosis of ASD, confirmed with the Autism Diagnostic Interview-Revised (ADI-R; *n* = 43; 15 female), and low-risk (LR) infants had an older sibling without ASD or language impairment (*n* = 45; 19 female); infants participated in a range of tasks at 6, 12, 18, and 24 months. Supplemental materials include information on inclusion/exclusion criteria (Additional file 2: Text S1.1, S1.2), full sample characteristics (Additional file 1: Table S1), measure-specific sample information for habituation (Additional file 1: Table S2), and ERP (Additional file 1: Table S3).

#### ASD assessment

At 24 months, a clinical best-estimate diagnosis was given for the HR group as defined in the DSM-IV [65] through the consensus judgment of a highly experienced certified clinical assessor and licensed clinical psychologist, based on all available information obtained through the ADOS, cognitive testing, parental interview (the ADI-R adapted for use with toddlers [66] and other developmental history information provided by the parent during testing sessions), and all other experiences with the infants. Based on this information, infants were classified according to the DSM-IV criteria as having “autistic disorder,” “pervasive developmental disorder—not otherwise specified,” or “no diagnosis.” Clinicians judged their confidence in the classification as “very confident,” “somewhat confident,” or “not confident.”

For analysis, infants within the HR group were divided based on consensus clinical judgment of their diagnostic outcome at 24 months. Of the original group of 43 HR infants, three did not receive a 24-month assessment and were not included in analyses. Infants in the ASD+ group (*n* = 12) were all judged to meet the DSM-IV criteria for either Autistic Disorder (*n* = 2) or Pervasive Developmental Disorder—Not Otherwise Specified (*n* = 10) at 24 months. Of note, in two cases where the clinician diagnostic classification was accompanied by a rating of “not confident,” confirmation of ASD group classification was supported by meeting on the ADOS algorithm. One of these infants met the cutoff for autism on the ADOS and was included in the ASD group; the second infant did not meet cutoff for ASD on the ADOS (ADOS total score = 5) and was excluded from outcome analyses. Infants in the HR-ASD-Neg group were judged to have “no diagnosis” on the clinical best-estimate DSM-IV criteria (*n* = 27). Additional file 2: Text S1.3. provides further information about ASD assessment procedures; Additional file 1: Table S1 shows diagnostic and developmental information for all groups.

The majority of low-risk controls did not receive an in-person assessment at 24 months. However, parents of a subset of *n* = 22 completed the Social Communication Questionnaire when their children were an average age of 44 months (range 37–64 months). All 22 infants scored below 11 on this instrument. Further, all 22 of these infants were judged at 18 months to not have ASD (with a rating of “very confident”) by a team of experienced clinicians, and all 22 had Vineland Socialization and Communication scores within the typical range at age 24 months. Thus, we are confident that this group of infants did not have ASD. Analyses below include only this subset of *n* = 22 LR-ASD-Neg infants. However, all patterns of significance presented remain the same if the whole LR group is included.

#### Analysis strategy

Our primary research questions relate to ASD Outcome. Thus, we primarily compared the differences between infants who did (ASD, *n* = 12) and did not (ASD-Neg, *n* = 49) later develop ASD, collapsed across risk groups. Several previous studies have also used the strategy of contrasting ASD with no ASD outcome collapsed across risk group [11, 67–72]. To verify that there were no effects of familial risk status within the ASD-Neg group, we examined high versus low familial risk (HR-ASD-Neg versus LR-ASD-Neg). These analyses did not reveal any differences on key variables, confirming the validity of our approach.

Because of the low overlap between infants with data at both 6 and 12 months for the ERP task, across both habituation and ERP tasks, we first present analyses of age groups separately for comparability between measures. For the habituation task only, we subsequently present analysis of data from infants who contributed data at both 6 and 12 months.

#### Habituation

The method exactly replicated that described in Experiment 1. Valid data was obtained from 84 % of 6-month-old infants and 98 % of 12-month-old infants (see Additional file 1: Table S2 for full details of inclusion rates by group). Habituation variables were analyzed in two repeated-measures ANOVAs on peak look duration and peak look position separately, both by group (ASD+, ASD-Neg), gender (male, female), and stimulus (face, toy).

For dishabituation, as in experiment 1, we first used repeated-measures ANOVAs on looking times to the familiar versus novel stimulus by group (ASD-Neg, ASD+) and gender (male, female) for each age (6, 12 months), stimulus (face, object), and delay (short, long) condition separately. A significant effect of familiar versus novel indicates dishabituation in that paradigm; significant interactions with group would indicate that dishabituation magnitudes differed by group.

We then analyzed longitudinal effects with data from infants who had sufficient data at both time-points (*n* = 51 infants; *n* = 42 ASD-Neg, *n* = 9 ASD+). Specifically, we used repeated-measures ANOVA on peak look duration and peak look position by age (6, 12 months), group (ASD+, ASD-Neg), gender (male, female), and stimulus (face, object). We also examined longitudinal change in dishabituation parameters using repeated-measures ANOVA on look duration by familiarity (familiar, novel), age (6, 12 months), group (ASD+, ASD-Neg), and gender (male, female) for each stimulus (face, object) and delay (short, long) condition separately to maximize participant inclusion. Age-related group differences in dishabituation would be reflected in interactions between age (6, 12 months), group (ASD+, ASD-Neg), and familiarity (familiar, novel).

Finally, to identify whether infants with later ASD show a distinctive pattern of age-related change in peak look duration or peak look position, we also performed a cluster analysis on change scores between 6 and 12 months for the face and object habituation tasks in the whole group of infants (*n* = 51) and examined the outcome status of infants that fell within these clusters using a chi-squared analysis (following [22, 73]).

#### Event-related potential task

The method exactly replicated that described in experiment 1. Valid data was obtained from 55 % of 6-month-old infants and 53 % of 12-month-old infants. Additional file 2: Text S1.5. and Additional file 1: Table S3 give further details of data inclusion and exclusion rates for the longitudinally assessed infants. Importantly, there were no significant differences in the final number of *attended*, *artifact-free trials* included in analysis between outcome groups or stimulus categories at either age (*F*s < 1.5, *p*s > .2; see Additional file 1: Table S3).

P400 data was analyzed in two repeated-measures ANOVAs on P400 latency and amplitude separately, both by age (6, 12 months), group (ASD+, ASD-Neg), laterality (left, right), and stimulus (face, toy). Gender was not included as a factor due to the small size of the ASD+ group. Nc data was analyzed in three repeated-measures ANOVAs on Nc duration, Nc early, and Nc late components separately, all by age (6, 12 months), group (ASD+, ASD-Neg), and stimulus (face, toy).

##### Correlations with behavior

Finally, within the high-risk group, we correlated key experimental variables (mean peak look duration to faces and objects at 6 months, P400 latency to faces over the left and right hemispheres, and late Nc amplitude) with key behavioral variables (Mullen verbal and nonverbal standard scores collected concurrently with experimental variables; and ADOS total scores at 24 m) using Pearson’s correlation coefficients. Relations with concurrent behavioral variables may indicate confounds of general developmental level; predictive relations with later ADOS scores within the high-risk group as a whole would strengthen results from analysis by categorical outcome group. We predicted that there would be no significant concurrent relations with developmental level but that there would be predictive relations to later ADOS scores.

## Results

### Habituation to faces and objects by ASD outcome

#### 6 months

##### Peak look duration

In a repeated-measures ANOVA on peak look duration by gender (male, female), group (ASD+, ASD-Neg), and stimulus (face, object), infants showed longer peak looks to faces than objects (*F*(1,50) = 4.13, *p* = 0.05, *η*^2^ = 0.08). This pattern is consistent with the normative data shown in Experiment 1 and previous work [63]. However, as illustrated in Fig. 1b, 6-month-old infants who later met the DSM-IV criteria for ASD at 24 months (ASD+ *n* = 9) showed significantly *shorter* peak look durations in face and object habituation tasks than infants who did not meet criteria for ASD (ASD-Neg; *n* = 43, main effect of outcome group: *F*(1,48) = 4.11, *p* = 0.04, *η*^2^ = 0.08; Fig. 1b).

##### Peak look position

In a repeated-measures ANOVA on peak look position by gender (male, female), group (ASD+, ASD-Neg), and stimulus (face, object), there was an interaction between group and stimulus (*F*(1,48) = 5.99, *p* = 0.018, *η*^2^ = 0.11). Follow-up ANOVAs on peak look position for faces and objects separately by group (ASD+, ASD-Neg) showed that the ASD+ group produced a later peak look than the ASD-Neg group for faces (*F*(1,50) = 3.97, *p* = 0.05, *η*^2^ = 0.07; Fig. 1e) but not for objects, where if anything, the peak look for the ASD+ group was slightly earlier than the ASD-Neg group (*F*(1,50) = 2.15, *p* = 0.15, *η*^2^ = 0.04).

##### Dishabituation

In a series of repeated-measures ANOVAs on looking time by familiarity (novel, familiar), gender (male, female), and group (ASD, ASD-Neg) for each stimulus (face, object) and delay condition (short, long) separately, infants significant dishabituated to a within-category novel stimulus in all tasks (*F*s > 4, *p*s < 0.05), with no differences between outcome groups in the magnitude of dishabituation (see Additional file 1: Table S4), indicating that differences observed during habituation do not reflect a failure to learn about the stimuli (*F*s < 2, *p*s > 0.15).

#### 12 months

##### Peak look duration

In a repeated-measures ANOVA on peak look duration by gender (male, female), group (ASD, ASD-Neg), and stimulus (face, object) at 12 months (ASD+ *n* = 12; ASD-Neg *n* = 47), there were no significant outcome group differences for peak look (*F*(1,55) = 0.77, *p* = 0.39, *η*^2^ = 0.01; Fig. 1c) and no significant effect of stimulus (*F*(1,55) = 0.31, *p* = 0.58, *η*^2^ = 0.006).

##### Peak look position

In a repeated-measures ANOVA on peak look position by gender (male, female), group (ASD, ASD-Neg), and stimulus (face, object), there was again no significant effect of outcome group (group: *F*(1,55) = 0.3, *p* = 0.59, *η*^2^ = 0.005; stimulus by group: *F*(1,55) = 0.27, *p* = 0.61, *η*^2^ = 0.005; Fig. 1f), indicating that these effects were more pronounced in early development (Fig. 1b, d, e). Of note, the effect sizes for these analyses indicate that a sample size of at least 788 infants would be required to have 80 % power of such effects being significant.

##### Dishabituation

In a series of repeated-measures ANOVAs on looking time by familiarity (novel, familiar), gender (male, female), and group (ASD, ASD-Neg) for each stimulus (face, object) and delay condition (short, long) separately, infants significantly dishabituated to a within-category novel stimulus in all tasks (*F*s > 20, *p*s < 0.001), with no significant differences between outcome groups in the magnitude of dishabituation (*F*s < 3, *p*s > 0.09; see Additional file 1: Table S4), indicating that differences observed during habituation do not reflect a failure to learn about the stimuli.

#### Longitudinal analysis

##### Peak look duration

In infants who provided sufficient longitudinal data (*n* = 9 ASD+, *n* = 42 ASD-Neg), a repeated-measures ANOVA on peak look duration by age (6, 12 months), group (ASD+, ASD-Neg), gender (male, female), and stimulus (face, object) showed a significant interaction between age (6, 12 months) and group (ASD+, ASD-Neg); *F*(1,47) = 4.24, *p* = 0.045, *η*^2^ = 0.083). This confirms that group differences were significantly stronger at 6 months than 12 months. There was also a marginally significant interaction between age (6, 12 months) and stimulus (face, object); *F*(1,47) = 3.71, *p* = 0.06, *η*^2^ = 0.073) such that there were longer peak looks to faces than objects at 6 months but not 12 months (6 months face M 20.46, SE 2.92; object M 14.87, SE 1.78; 12 months face M 14.23, SE 1.41; object M 14.77, SE = 1.04).

Follow-up ANOVAs by age (6, 12 months) and stimulus (face, object) for each group separately showed that peak look durations were shorter at 12 months than 6 months for the ASD-Neg group (*F*(1,41) = 19.41, *p* < 0.001, *η*^2^ = 0.032; 6 m *M* = 21.97, *SE* = 1.76; 12 m *M* = 14.4, *SE* = 0.79) but not the ASD+ group (*F*(1,8) = 0.1, *p* = 0.76, *η*^2^ = 0.01; 6 m *M* = 14.08, *SE* = 1.29; 12 m *M* = 14.62, *SE* = 1.31). These results confirm that there were age-related decreases in habituation times to faces and objects for the ASD-Neg group that were not present for the ASD+ group.

##### Peak look position

A repeated-measures ANOVA on peak look position by age (6, 12 months), group (ASD+, ASD-Neg), gender (male, female), and stimulus (face, object) showed a significant interaction between stimulus (face, toy) and group (ASD+, ASD-Neg); *F*(1,47) = 4.32, *p* = 0.043, *η*^2^ = 0.084) and a marginally significant interaction between age (6, 12 months), stimulus (face, toy), and group (ASD+, ASD-Neg); *F*(1,47) = 3.40, *p* = 0.07, *η*^2^ = 0.067). Follow-up ANOVAs by age (6, 12 months) and stimulus (face, object) for each group separately showed a marginally significant interaction between age (6, 12 months) and stimulus (face, object) in the ASD+ group such that peak looks were later to faces than objects at 6 versus 12 months (*F*(1,8) = 4.53, *p* = 0.06, *η*^2^ = 0.36; 6 m face *M* = 4.61, *SE* = 1.14; 12 m face *M* = 2.28, *SE* = 0.36; 6 m object *M* = 1.94, *SE* = 0.54; 12 m object *M* = 2.72, *SE* = 0.59). There was no significant interaction in the ASD-Neg group (*F*(1,41) = 0.07, *p* = 0.80, *η*^2^ = 0.001; 6 m face *M* = 2.81, *SE* = 0.36; 12 m face *M* = 2.54, *SE* = 0.20; 6 m object *M* = 3.33, *SE* = 0.42; 12 m object *M* = 2.87, *SE* = 0.35). Although with limited power, these results broadly confirm the results reported in the cross-sectional sample, such that effects of ASD outcome were greater at 6 months than 12 months.

##### Dishabituation

A repeated-measures ANOVA on look durations by age (6, 12 months), group (ASD+, ASD-Neg), gender (male, female), and familiarity (novel, familiar) for each stimulus (face, object) and delay (short, long) category separately showed significant dishabituation (*F*s > 18, *p*s < 0.001) that did not interact with age, group, or gender for the two face habituation tasks and for the toy long condition (*F*s < 3, *p*s > 0.1). For the toy short condition, there were both a significant effect of familiarity (*F*(1,34 = 26.74, *p* < 0.001, *η*^2^ = 0.44) and an interaction between familiarity (novel, familiar) and age (*F*(1,34) = 7.1, *p* = 0.012, *η*^2^ = 0.17) such that dishabituation magnitude was larger at 12 months (novel *M* = 12.36, *SE* = 1.80; familiar *M* = 3.73 *SE* = 0.71) than 6 months (novel *M* = 6.27, *SE* =1.52; familiar *M* = 3.28 *SE* = 0.72). However, there were no interactions involving group and familiarity (*F*s < 2.5, *p*s > 0.1). Thus, there was no evidence of group differences that varied by age in dishabituation magnitude.

#### Cluster analysis

To establish whether the pattern of habituation variables observed in the ASD+ outcome group represents a distinct cluster within the group of infants as a whole, we performed a two-step cluster analysis using the change in peak look duration to faces and objects between 6 and 12 months, and the difference between peak look duration to faces and toys at 6 months, as input variables. This produced three clusters, showing a good fit to the data (illustrated in Additional file 1: Figure S4). Additional file 1: Table S6 shows habituation and clinical variables for each cluster. Briefly, cluster 1 (*n* = 14, *n* = 0 ASD+) showed a large decrease in peak look duration between 6 and 12 months and a slightly earlier peak look to faces than toys at 6 months. Cluster 2 (*n* = 31; *n* = 5 ASD+) showed a smaller decrease in peak look duration between 6 and 12 months and an earlier peak look to faces than toys at 6 months. Cluster 3 (*n* = 6; *n* = 4 ASD+) showed no decrease in peak look duration between 6 and 12 months to either faces or objects and a substantially later peak look to faces than objects at 6 months. A chi-squared analysis showed that there was a significant difference between the number of children falling into the ASD+ and ASD-Neg groups across clusters (*χ*^2^(50) = 14.3, *p* = 0.001). We then explored whether infants with ASD who fell into clusters 2 and 3 differed from each other using a series of univariate ANOVAs by cluster on Mullen verbal and nonverbal scores, Vineland Socialization and Communication standard scores, and ADOS total scores at 24 months (see Additional file 1: Table S6). Infants with ASD in cluster 3 showed generally poorer functioning levels at 24 months than infants with later ASD in cluster 2; analysis showed significant differences in Vineland socialization scores (*F*(1,8) = 15.9, *p* = 0.007, *η*^2^ = 0.73) and ADOS total scores (*F*(1,8) = 6.23, *p* = 0.047, *η*^2^ = 0.51) were significantly poorer in cluster 3 versus cluster 2 for infants with later ASD. These results are broadly consistent with previous work that has identified clusters of infants who do not show changes in peak look over the first year and who have poorer outcomes later in development [73].

### Neural responses to faces and objects by ASD Outcome

#### 6 months

##### P400 latency

In a repeated-measures ANOVA on P400 latency by outcome group (ASD-Neg *n* = 25, ASD+ *n* = 6), stimulus (face, object), and laterality (left, right), there was a main effect of stimulus such that P400 latencies were faster to faces than objects (*F*(1,29) = 7.32, *p* = 0.011, *η*^2^ = 0.20); this resembles the normative pattern seen in experiment 1. However, there was a significant interaction between stimulus and outcome group (*F*(1,29) = 10.8, *p* = 0.003, *η*^2^ = 0.27) such that for the face condition only, the P400 peaked significantly *earlier* in the ASD+ group than the ASD-Neg group (*F*(1,29) = 5.74, *p* = 0.023, *η*^2^ = 0.17; Fig. 2b). There was also a significant interaction between stimulus and laterality (*F*(1,29) = 4.15, *p* = 0.05, *η*^2^ = 0.13) and stimulus, laterality, and outcome group (*F*(1,29) = 4.74, *p* = 0.038, *η*^2^ = 0.14). Figure 2b illustrates this interaction: effects of group were strongest for faces over the left hemisphere.

##### P400 amplitude

In a repeated-measures ANOVA on P400 amplitude by outcome group (ASD-Neg *n* = 25, ASD+ *n* = 6), stimulus (face, object), and laterality (left, right), there were no significant effects of outcome group on P400 amplitude (*F*s < 2.5, *p*s > 0.15).

To check whether these effects could reflect a follow-on effect from changes in the P1 and N290, we examined group differences in the amplitude and latency of these components; none were significant (see Additional file 2: Text S2.4).

##### Nc amplitude

**Fig. 3:**
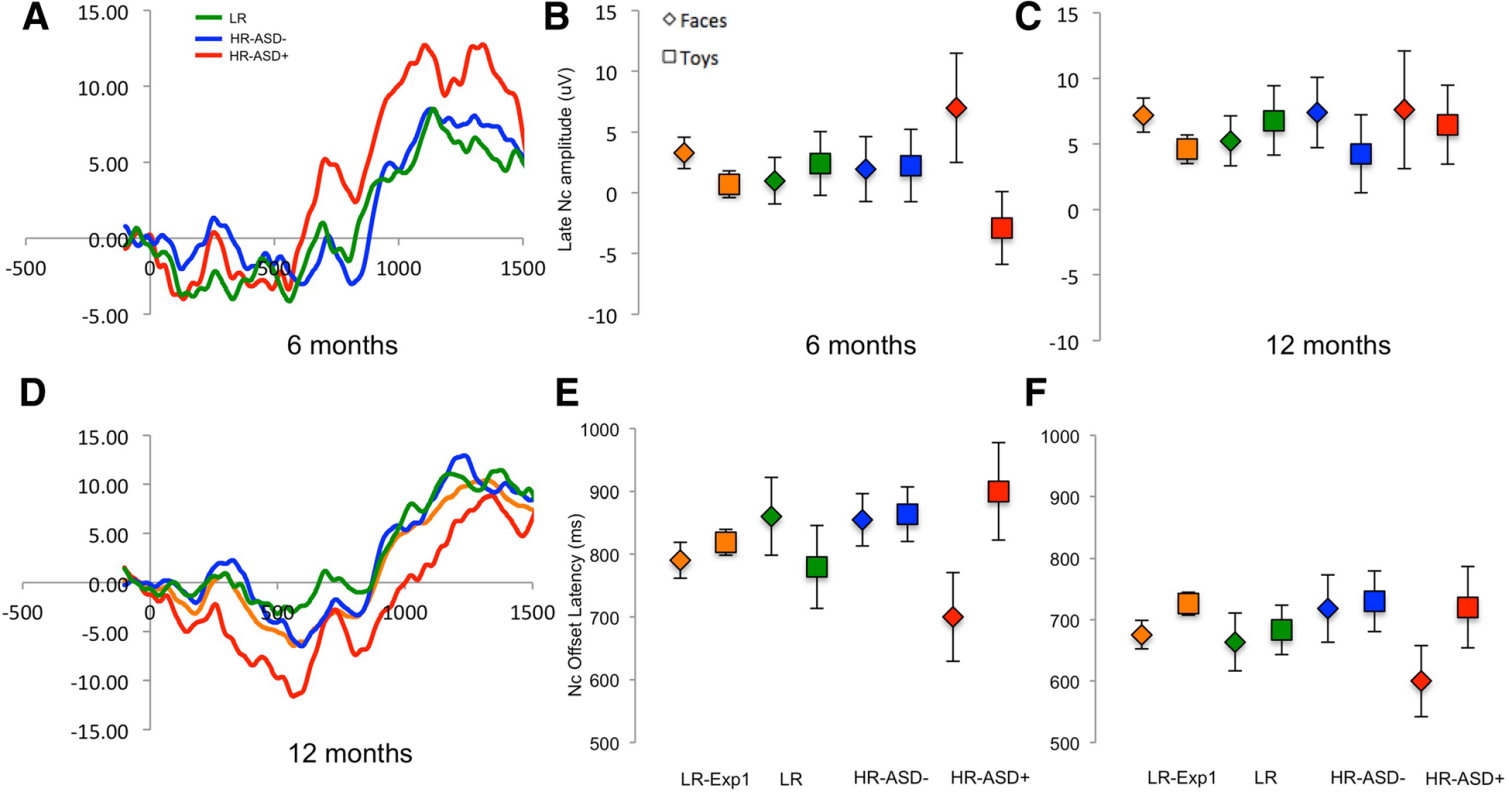
Anterior event-related potentials. **a** Illustration of the grand average event-related potential over frontal electrodes at 6 months. **b** Illustration of the grand average event-related potential over frontal electrodes at 12 months. **c** Mean amplitude of the late Nc component to faces or objects at 6 months in the three groups (low-risk control, HR-ASD+, and HR ASD-Neg). **d** Mean amplitude of the late Nc component to faces or objects at 6 months. **e** Mean offset latency of the late Nc component to faces or objects at 6 months. **f** Mean amplitude of the late Nc component to faces or objects at 12 months. All error bars are ±1 standard error

For the Nc overall amplitude, repeated-measures ANOVA on early and late Nc mean amplitude by outcome group (ASD-Neg *n* = 25, ASD+ *n* = 6) and stimulus (face, object) showed no significant effects on the early Nc (*F*s < 2.5, *p*s > .15). However, for the late Nc, there was a significant interaction between stimulus and outcome group (*F*(1,29) = 5.84, *p* = 0.022, *η*^2^ = 0.17; Fig. 3b). Overall, the ASD+ group had a more negative Nc component to objects than faces, while the ASD-Neg group did not.

##### Nc duration

For Nc duration, repeated-measures ANOVA on early and late Nc mean amplitude by outcome group (ASD-Neg *n* = 25, ASD+ *n* = 6) and stimulus (face, object) showed that there was a significant interaction between stimulus and outcome group (*F*(1,24) = 4.2, *p* = 0.05, *η*^2^ = 0.15; Fig. 3e). The ASD+ group showed a faster Nc offset to faces than objects, while the ASD-Neg group did not.

#### 12 months

Again suggesting that these effects were most pronounced in early development, there were no significant effects of outcome group on P400 latency at 12 months (ASD-Neg *n* = 26, ASD+ *n* = 5; stimulus by group *F*(1,29) = 0.55, *p* = 0.47, *η*^2^ = 0.02; Fig. 2c), late Nc amplitude (stimulus by group *F*(1,29) = 0.25, *p* = 0.62, *η*^2^ = 0.008; Fig. 3c), or for other comparisons (*F*s < 3, *p*s > 0.1). Power analysis based on effect sizes indicates that a minimum of at least 370 infants would be required for effects of this magnitude to reach significance.

As can be seen in Fig. 3f, the effects of Nc offset latency were not significant but were in the same direction at 12 months as 6 months, suggesting the developmental change was less clear on this metric (*F*(1,23) = 2.51, *p* = 0.13, *η*^2^ = 0.09).

Of note, only 16/49 ASD-Neg infants and 2/12 ASD+ infants were included at both age points. To determine whether the different patterns of effects at 12 and 6 months were due to differences in the children included at each age, we examined whether there were any significant differences in 24-month ADOS scores or 6 and 12-month Mullen composite scores between the groups of infants included in analyses at 6 and 12 months. These analyses are presented in Additional file 2: Text (S2.3); to summarize, infants with data at 12 months only showed lower Mullen scores and higher ADOS scores at 24 months (but not 6 or 12 months) than infants with data at 6 months only or infants with longitudinal data. If anything, this would be expected to magnify differences at 12 months (since infants at that age were most impaired), which does not match our pattern of findings.

### Correlations with behavior

**Fig. 4:**
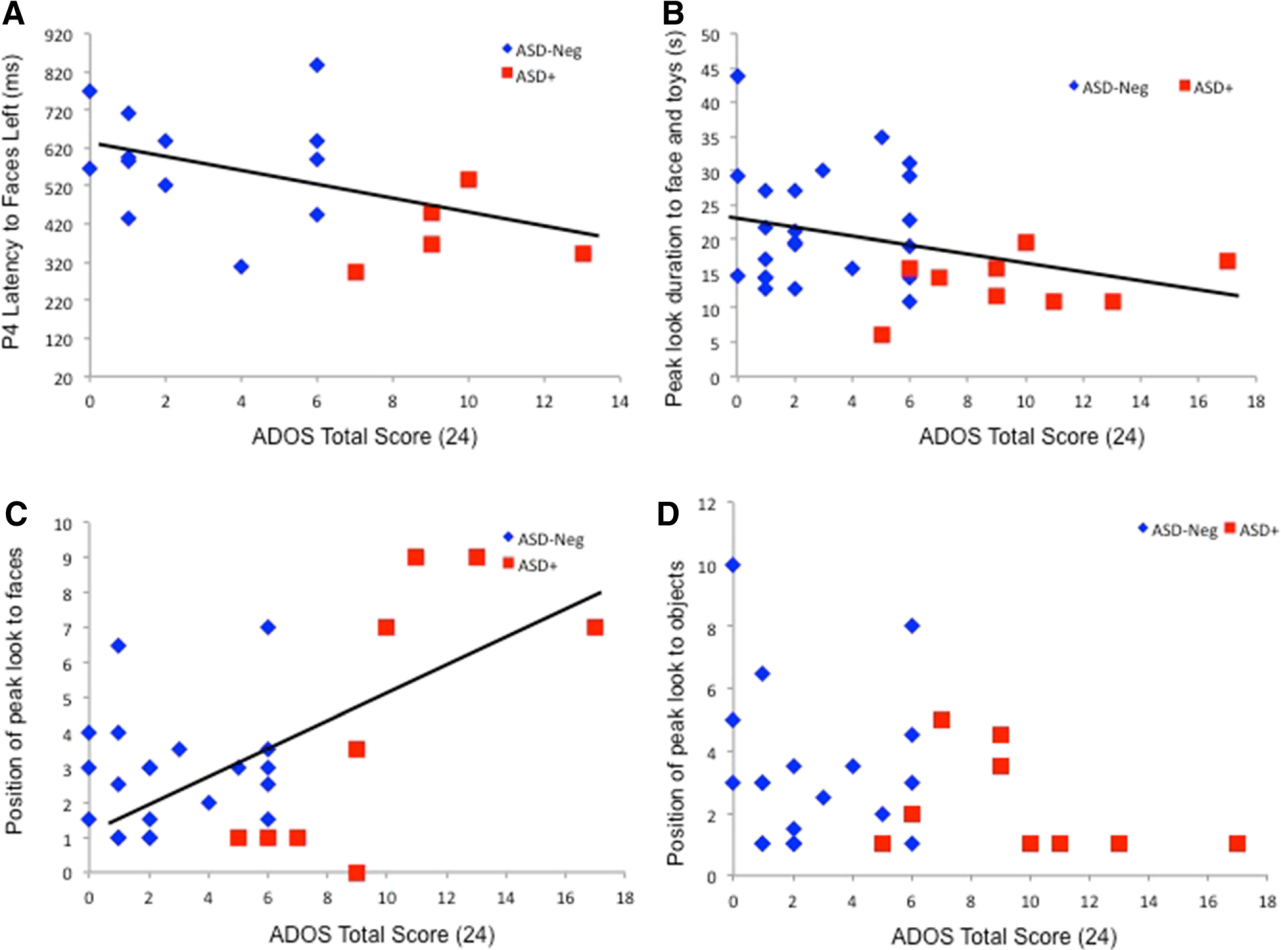
Relations between 24-month ADOS scores and attention engagement at 6 months in high-risk infants. **a** Relation between shorter P400 latencies to faces over the left hemisphere and higher ADOS total score at 24 months. **b** Relation between shorter peak look duration to faces and objects and higher ADOS total scores at 24 months. **c** Relation between later peak look to faces and higher ADOS total scores at 24 months. **d** No significant relation between later peak look to objects and ADOS scores at 24 months

Within the high-risk group, we correlated key experimental variables (mean peak look duration to faces and objects at 6 months, P400 latency to faces over the left and right hemispheres, and late Nc amplitude) with key behavioral variables (Mullen verbal and nonverbal standard scores collected concurrently with experimental variables; and ADOS total scores at 24 m). There were no significant correlations between experimental variables and concurrent behavioral variables, confirming that results were not confounded with concurrent developmental level (*r*s < .25, *p*s > 0.07). However, higher 24-month ADOS total scores were significantly correlated with later peak look position to faces at 6 months (*r*(30) = 0.58, *p* = 0.001), shorter P4 latency to faces over the left hemisphere (*r*(18) = −0.46, *p* = 0.05), and marginally significantly with shorter mean peak look duration to faces and objects at 6 months (*r*(32) = −0.32, *p* = 0.08). These relations are illustrated in Fig. 4. Taken together, these results support categorical analyses in suggesting that shorter epochs of attention to social stimuli were related to later autistic symptomatology.

#### Summary

Significant differences in our target habituation parameters were observed between infants who did (ASD+) and did not (ASD-Neg) meet criteria for ASD at 24 months. Specifically, 6-month-old infants with later ASD showed *shorter* look durations than other infants, and their peak look to faces (but not objects) was observed later in the habituation function than for other infants (Fig. 1). Delayed peak look to faces was also continuously related to higher ASD symptoms at 24 months within the ASD group as a whole (Fig. 4). Taken together, these findings suggest disruption to early metrics of social attention and learning in the ASD+ group. Further, none of these effects were apparent at 12 months, suggesting that they are most pronounced in early infancy. This was unlikely to be due to limited power since effect sizes for the 12-month comparisons were small (*d* < 0.2) and the sample size was slightly larger than for the 6-month analysis. In the longitudinal analysis, significant interactions between age and outcome group were observed for peak look duration, confirming that effects were stronger at 6 months than 12 months. Further, a preliminary cluster analysis across all infants with longitudinal data (*n* = 51) showed that infants with ASD were disproportionately represented within a cluster who showed limited age-related change in peak look to faces and objects and delayed peak look to faces at 6 months. This is consistent with previous work that has identified clusters of “non-normative” typically developing infants who have poorer developmental outcomes [73]. Examination of the ASD-Neg group by risk status indicated patterns of stimulus and age effects that matched those seen in our normative data in experiment 1 (see Fig. 1) and revealed no differences between infants with different levels of familial risk. Taken together, these findings support the hypothesis that attention engagement is atypical at 6 months in infants with later ASD. Furthermore, infants at high familial risk who do not develop ASD show attention engagement patterns consistent with low risk typically developing infants.

For ERP measures, we observed significantly *faster* P400 latencies to faces in the ASD+ group (see Fig. 2b), particularly over the left hemisphere. Further, the Nc was smaller and shorter to faces versus objects in the ASD+ versus ASD-Neg groups. These three variables (shorter P400 latency to faces over the left hemisphere, shorter and smaller Nc to faces) were also significantly associated at 6 months in our typically developing normative group (Experiment 1). Shorter P400 latencies to faces were also continuously related to later ASD symptoms within the high-risk group as a whole, mitigating the small sample size of infants with later ASD. Thus, this pattern appears to reflect a cohesive picture in which infants with later ASD show reduced attention capture by faces. As for the habituation results, these effects were not observed at 12 months, suggesting that they are most pronounced in early infancy.

## Discussion

In this study, we used multiple techniques to assess fundamental aspects of social and nonsocial processing development in a large sample of low-risk infants and a longitudinal sample of infants at high and low risk for ASD. Results showed that high-risk infants with later ASD demonstrate neural and cognitive differences that are most pronounced for social stimuli. Six-month-old infants at high-risk for ASD who met DSM criteria for ASD at 24 months showed (1) significantly *shorter* peak look durations during a habituation paradigm, which may signal disruptions in sustained attention; (2) significantly *delayed* peak looks to faces during habituation, suggesting disrupted or delayed engagement of sensitization to social stimuli, (3) significantly *faster* P400 responses to faces; and (4) a smaller and shorter Nc response to faces, which may represent less sustained neural responses to faces. These effects were not detected at 12 months, indicating that these markers might be specific to early infancy. This study represents the first demonstration of early differences in depth of social attention contrasted with effects on nonsocial stimuli and are supported by both behavioral and neural data. Our results are also consistent with neuroconstructivist approach to development [48, 74] and suggest that early brain and behavioral development in infants who go on to develop ASD is dynamic, and risk markers may change rapidly over the course of early development [21].

### Differences in social attention in infants with later ASD

Habituation paradigms are the most widely used method of assessing visual attention in infancy [33]. Consistent with previous work, in the present study, a normative sample of low-risk typically developing infants and infants from low and high-risk groups who did not later develop ASD showed a longer peak look to faces than objects [63], decreased peak look duration over the first year [75], and dishabituated to a novel face or an object after delays of up to a minute [76]. However, infants who developed ASD showed a shorter peak look that was later in the habituation function for faces than objects. The “dual-process” account [32] of habituation argues that the position of a peak look in the sequence reflects a process of “sensitization” that is associated with a spike in parasympathetic arousal that *increases* attention to the stimulus [33]. The later peak look to faces observed in infants with later ASD (that was also continuously related to poorer social functioning across the high-risk group as a whole) may thus indicate that disruptions to sensitization are associated with later ASD symptoms. Sensitization is thought to be important in engaging deeper levels of processing and has been implicated in the facilitation of learning by infant-directed speech [34, 35]. Disrupted sensitization could have a negative impact on social development. Interestingly, a longitudinal study of typically developing infants revealed a cluster who showed rapid habituation times that did not decreases with age; these infants showed atypical developmental decreases in sensitization (reflected in heart rate deceleration) and poor language skills at 24 months [73, 77]. Although preliminary, a similar cluster was observed in the present dataset and contained a disproportionate number of infants with later ASD. These infants also showed poorer social functioning at outcome than infants with later ASD with other patterns of early habituation data. Shorter peak looks and the delayed peak look to faces in infants with later ASD could thus reflect altered timing of the physiological sensitization response to social stimuli, and thus delayed or disrupt engagement of deeper levels of attention.

The faster P400, and smaller and less sustained Nc ERP response, may also reflect a reduced depth of processing for social stimuli. The Nc component has been extensively studied in infancy. Because the Nc is modulated by novelty and stimulus salience [37], and is larger (more negative) to stimuli presented during physiologically defined states of attention [39], the Nc is thought to reflect attention engagement [40]. Thus, the smaller and shorter Nc observed in infants with later ASD would be expected to reflect reduced attention engagement to faces. The function of the P400 in infancy is less clear but may relate to semantic aspects of extracting information from faces [40]. Results with typically developing infants in experiment 1 indicate that faster P400 latency relates to a smaller and shorter Nc, consistent with our hypothesis that a faster P400 latency to faces may reflect reduced depth of processing. Other work with toddlers and young children with ASD has also noted atypicalities in both the P400 and Nc response to faces. For example, toddlers and young children with ASD show developmentally delayed modulation of the Nc by facial familiarity, such that responses resemble those seen in younger typically developing toddlers [27]. The same group of toddlers with ASD also show atypical modulation of the P400 by facial familiarity; specifically, typically developing toddlers showed a larger P400 amplitude to unfamiliar than familiar faces, while toddlers with ASD did not [41]. Further, the same group of children with ASD tested at age 4 to 5 years showed a slower Nc peak latency to faces than objects, which was reversed in children from that group who had received 2 years of intensive treatment that improved social functioning [59]. Thus, our results in infancy are consistent with the sensitivity of the P400 and Nc ERP components to atypical social attention in the early development of children with ASD. Future work should determine whether our infant attention metrics are also sensitive to the effects of early intervention [78].

Our interpretation of reduced depth of social attention engagement is consistent with other works. For example, Chawarska and colleagues [17, 18] have observed reduced monitoring of social scenes at 6 months. This is particularly apparent when faces were accompanied by speech, suggesting that deficits may be exaggerated during more complex naturalistic presentations. Further, Jones and Klin [21] noted a declining pattern of attention to the eyes of faces between 2 and 6 months, suggesting an emerging profile of disrupted social attention. Finally, Wass and colleagues [79] noted shorter durations of individual fixations during scanning of complex scenes (although comparison of fixations to faces versus non-faces did not reach significance). The present study adds to this literature by showing that there are atypicalities not only in the direction of visual attention but also in its temporal dynamics. Taken together, there is mounting evidence that there are early disruptions in the depth, direction, and quality of social attention in the first 6 months of life that precede the emergence of clear behavioral symptoms of ASD. Such disruptions could reduce the quality of social information processing, leading to cascading deficits in social behaviors that emerge over time [6].

However, other studies have not noted differences in social attention in infants with later ASD. For example, Ozonoff and colleagues [11] found that infants with later ASD showed a typical number of gazes to faces per minute during a cognitive task at 6 months, and Elsabbagh and colleagues [19, 20] found normal rapid orienting to a face in a complex array and normal shifting of attention between the eyes and mouth of a person telling nursery rhymes. One potential explanation stemming from the present study requires us to differentiate between processes of attention orienting and attention maintenance (variously called sustained attention or attention holding), which are subserved by different neural systems [80]. Typically, physiologically defined states of sustained attention follow an initial orienting response, and only emerge 1 to 2 s after a period of looking to a stimulus begins [81]. It may be that attention orienting to social stimuli is relatively typical at 6 months in infants with later ASD [82], but attention maintenance is disrupted. The present study is consistent with this pattern: we observed atypicalities in the duration and quality of attention, while controlling initial attention capture (since stimuli were presented while infants were looking at the screen). Further, atypicalities were noted in the late but not early Nc components, consistent with this hypothesis. Studies that measure only the number of gazes to a face per minute [11] and studies that use complex arrays that elicit very short individual fixations of average duration 600 ms [19] may not detect overall deficits in attention allocation. In contrast, studies that measure overall monitoring of a social stimulus over a longer period [17, 18] may be more sensitive to the accumulated effects of shorter individual attention epochs. If this explanation is correct, future work should contrast attention orienting and attention engagement within the same paradigm with infants with later ASD; one valuable approach would be to use heart rate-defined phases of attention to separate effects on orienting and attention maintenance.

### Age-related changes in social attention

Six-month-old high-risk infants who later met criteria for ASD showed significantly *shorter* peak looks to faces than high-risk infants without early ASD. Dishabituation was unaffected, indicating that shortened habituation times did not reflect failure to learn about the stimulus. Rather, infants with later ASD showed a similar magnitude of dishabituation despite significantly shorter habituation times. These results are in apparent contrast to previous work showing *increased* habituation times to faces versus houses in clinically-referred toddlers with an ASD diagnosis [22]. What could account for this disparity? In the study by Webb and colleagues, prolonged habituation times were only apparent in those with the highest level of symptoms (ADOS total scores over 17). Consistent with other work with high-risk infants, the present sample tended to be more mildly affected: indeed, only one child had an ADOS score over 17 at 24 months. Possibly, there is a complex relation between sustained attention and levels of ASD symptoms. Alternatively, developmental stage may be a critical factor, particularly since no group differences were observed in patterns of habituation at 12 months in the present study. Indeed, several recent studies of high-risk infants reveal opposite patterns of disruption to those seen in young children with ASD. For example, long-range underconnectivity has been observed in children with ASD [83], while others have found *over*connectivity in high-risk infants [84] and infants with later ASD [85, 86]. Further, while children with ASD show a longer latency pupillary constriction to a sudden increase in luminance [87], infants at high familial risk show a faster constriction [88]. Temperament trajectories also suggest that high-risk infants are less active than controls in infancy but more active than controls in toddlerhood [89]. Thus, it is possible that ASD-related disruptions to sustained attention are expressed differently in infancy and toddlerhood.

Of note, 12-month-old infants with typical development (Experiment 1) and those without later ASD (Experiment 2) showed a decrease in peak look durations with age. There are several interpretations of this finding. Possibly, normative decreases in peak look duration reflect increased encoding speed across the first year of life, rather than changes in attention. Factors driving the variance in look duration with age may thus differ from those driving variance in look duration between diagnosis groups. Alternatively, there may be decreases in attention engagement to simple static stimuli across the first year. Indeed, several studies show that while look durations to simple stimuli typically decrease, look durations to more complex dynamic stimuli increase across the first year (for review [90]). This may reflect decreasing attention capture by simple static stimuli with developmental time and highlights the importance of both studies with highly controlled stimuli and studies involving more complex naturalistic settings.

### Perceptual processing in ASD

One alternative explanation of our results should be considered. The duration of individual looks during a habituation paradigm is influenced not only by sustained attention but also by the speed at which infants encode the stimulus [30, 49]. Thus, one possible alternative explanation is that infants who go on to ASD at 6 months are actually more efficient at structural face encoding. The observed faster P400 latencies to faces in the ASD+ group could also be considered a reflection of more rapid perceptual processing, which may contribute to more rapid encoding. Increased efficiency of perceptual encoding could potentially be related to *greater* experience with faces in the early development of infants with later ASD. Indeed two reports have observed *greater* attention to faces or eyes in the first 6 months in infants with later ASD relative to low-risk typically developing infants [11, 21]. This related increase in experience with faces would provide the potential to support faster learning [91]. Subsequently, the progressive decrease in interest in faces seen in infants with later ASD over the second year of life may fail to reinforce this initially advanced developmental trajectory of the face recognition system, leading to the gradual emergence of relatively slower learning [22] and delayed neural correlates of face processing [27]. This is consistent with models that propose that the emergence of ASD reduces social interest, which in turn affects the experience-dependent process of social learning [15]. Examining the developmental inter-relation between naturalistic measures of social attention and neurocognitive measures of social processing in high-risk infants will provide a further test of this model.

Better encoding or perceptual processing would, however, not be consistent with a range of other findings from our study. First, this would not account for the finding of a shorter and smaller Nc response to faces, since the Nc has not typically been associated with basic aspects of perceptual processing. Rather, the Nc is widely accepted as an index of attention engagement [40]. The correlation between the less negative and shorter Nc component with a faster P400 response in our large normative cohort in experiment 1 is consistent with the findings of experiment 2, in which infants with later ASD showed both a faster P400 response to faces and a smaller and shorter Nc. Taken together, this data suggests that the shorter P400 latency to faces, reduced Nc duration and amplitude in infants with later ASD may together reflect reduced attention engagement to social stimuli. Of note, although our ERP findings by outcome group are preliminary because of the relatively small number of infants with later ASD, we also observed a continuous association between P400 latency to faces (not objects) and later ADOS scores within the large high-risk group as a whole, supporting this finding. Second, the finding of delayed sensitization to faces (significantly later peak look to faces at 6 months) in the present dataset is not consistent with faster perceptual processing or encoding. Delayed sensitization also did not only occur at the group level: there was a strong continuous relation between delayed sensitization to faces and higher later ADOS scores. This delayed sensitization is strongly suggestive of reduced or altered attention engagement with faces in infants with later symptoms of ASD. Further, a cluster of infants showed delayed peak look to faces, and shorter peak look to faces and objects. The infants with later ASD within this cluster (*n* = 4/6) were significantly more impaired than infants with later ASD who did not show this pattern. These results are not consistent with shorter peak looks reflecting a “strength.” Third, there were no differences in the latency of the P1 or N290 ERP components (see Additional file 2) between infants with and without later ASD, and these are the two components that have been most closely associated with perceptual aspects of face processing. No difference in the latency of P1 or N290 ERP components has also been reported in other cohorts of infants with later ASD [36]. If perceptual processing or encoding of faces were more rapid in infants with later ASD, differences would be expected on these components. Finally, while a range of other studies have observed altered attention to social stimuli in young infants with later ASD (e.g., [17, 18]), there have been no reports consistent with more efficient encoding or perceptual processing of face stimuli (though see [79, 92] for possible evidence of more efficient processing of nonsocial stimuli). Thus, the weight of evidence from this study and from the previous literature supports our interpretation of reduced attention engagement to social stimuli at 6 months.

### Caveats

One important discussion point is some apparent inconsistencies between the present ERP results and a previous study [36]. Elsabbagh and colleagues presented repeated pictures of intact and scrambled faces to 6- to 10-month-old HR infants. There were no group differences in P400 latency to static faces in relation to HR-ASD+ outcome, although infants with later ASD did show less sensitivity over the P400 component to gaze shifts in a second set of stimuli [36]. However, Elsabbagh and colleagues did observe a faster P400 latency to faces than noise stimuli in infants with later ASD that was not observed in other groups, and this is consistent with the current dataset. Thus, the modulation of P400 latency by social versus nonsocial content was consistent across studies, but the overall decrease in P400 latency to faces was only observed in the present report. One possible explanation is clearly the relatively small sample size included in both studies in relation to the range of functioning levels seen in children with ASD. A second possibility is that the difference in findings reflects a developmental shift in which effects are found in the younger sample in this study (*M* = 6.1 months, range 6 to 8) but not the slightly older sample included in Elsabbagh’s work (*M* = 7.9 months, range 6 to 10) or in our 12-month-old sample. Alternatively, infants with later ASD may also have a particular advantage in the more challenging task of processing the trial-unique stimuli used in the present study relative to the repeated pictures used by Elsabbagh and colleagues.

Important in our interpretations, the present study used two-dimensional static screen-based stimuli. This has been a common strategy in studies of face processing in older children and adults with ASD [22, 59, 93], and in young infants [44, 94], and allows more precise matching of stimuli between social and nonsocial conditions. However, it will be important to examine in future work whether such results generalize to more naturalistic social stimuli. In early infancy, EEG markers may show greater sensitivity to social stimuli in live or dynamic contexts [36, 95, 96], and a range of evidence suggests that infants learn more effectively from live than recorded stimuli (e.g., [97, 98]). Thus, examining aspects of attention engagement to live dynamic social and nonsocial stimuli will be an important step for future work.

We related infant markers to ASD diagnosis at age 2 years in order to examine early learning mechanisms that might underlie the early appearance of ASD symptoms. This has become an increasingly common strategy in work with high-risk infants [86, 99, 100]. Diagnostic stability of clinical diagnoses made at age 2 years is typically high [4, 101, 102]. However, the relationship between variance in diagnosis trajectory (e.g., the presence or absence of regression) [103–105] based on outcome age and the underlying attention mechanisms or neurocognitive differences is unclear. There continue to be dynamic changes in ASD diagnosis, and symptom expression across the lifespan and emphasis on very early mechanisms will allow the development of more targeted pre-diagnostic interventions for high-risk infants [78].

Lastly, an important limitation is the relatively small number of children with later ASD in the ERP analyses. Despite this smaller group, we had sufficient power to detect significant and complementary effects at 6 months within both methodologies employed. The ERP findings were significantly correlated with habituation variables at 6 months, suggesting that the two experimental measures were tapping related processes. The number of children who met later criteria for ASD was comparable with recent studies of infants with later ASD [18, 21, 86]. Several aspects of the present data are also consistent with previous work. (1) Elsabbagh and colleagues also observed a faster P400 to faces than phase-scrambled faces in 6-month-old infants with later ASD, which is replicated in the present dataset. (2) Wass and colleagues report shorter fixation durations during viewing of a static scene at 6 months [79], which is consistent in direction with the shorter peak looks observed in the present dataset. (3) The direction of effects observed in infants with later ASD across different ERP components (a faster P400 to faces, and a shorter and smaller Nc to faces) is consistent with the internal structure of ERP findings from the large sample of typically developing infants tested in experiment 1 (in which a faster P400 response to faces correlated with a shorter and smaller Nc to faces). (4) Data from the habituation paradigm are also consistent with reductions in social attention at 6 months reported by other groups [17, 18]. Thus, our results are supported by both internal and external validation approaches.

## Conclusions

Taken together, the present data suggest that reduced sensitization or engagement with social stimuli at 6 months may be an important developmental mechanism underpinning later ASD. This early emerging deficit in attention engagement may influence the experience-expectant process of learning about social stimuli, over time leading to later-emerging deficits in face processing and declines in social orienting. Further work examining the inter-relation between social processing, social attention, and social development in larger samples of high-risk infants will provide an important test of this model.

## Additional files

Additional file 1:
**Table S1.** Descriptive and demographic information for participants included in the full sample. **Table S2.** Summary of participant providing data for the habituation task. **Table S3.** Summary of number of participants providing data for the event-related potential task. **Table S4.** Summary of habituation variables by Age, Risk Group, Outcome Group and Stimulus. **Table S5.** Summary of ERP variables by Age, Risk Group, Outcome Group, and Condition. Figures are Mean (Standard Deviation). **Table S6.** Summary of habituation and clinical variables by Cluster of infants. **Figure S1.** Location of electrodes used in the analysis for the P400 (left) and Nc (right) components. **Figure S2.** Nc (A) and P400 (B) ERP components for the normative sample in Experiment 1. **Figure S3.** Three dimensional scatter plot showing the three empirically identified clusters of infants. Additional file 2: Supplementary text concerning methods and results.

## References

[1] Center for Disease Control. (2014). at <http://www.cdc.gov/ncbddd/autism/data.html>

[2] American Psychiatric Association. *Diagnostic and statistical manual of mental disorders*. (Arlington: American Psychiatric Publishing, 2013).

[3] World Health Organization. The ICD-10 classification of mental and behavioral disorders: diagnostic criteria for research. (1993).

[4] Lord C (2006). Autism from 2 to 9 years of age. Arch Gen Psychiatry.

[5] Herlihy L, Knoch K, Vibert B & Fein D. Parents’ first concerns about toddlers with autism spectrum disorder: effect of sibling status. *Autism* 1362361313509731 (2013). doi:10.1177/1362361313509731 10.1177/1362361313509731 PMC4386719 24216070

[6] Jones EJH, Gliga T, Bedford R, Charman T, Johnson MH (2014). Developmental pathways to autism: a review of prospective studies of infants at risk. Neurosci Biobehav Rev.

[7] Ozonoff S (2011). Recurrence risk for autism spectrum disorders: a Baby Siblings Research Consortium study. Pediatrics.

[8] Dawson G, Osterling J, Meltzoff AN, Kuhl P (2000). Case study of the development of an infant with autism from birth to two years of age. J Appl Dev Psychol.

[9] Werner E, Dawson G, Osterling J, Dinno N (2000). Brief report: recognition of autism spectrum disorder before one year of age: a retrospective study based on home videotapes. J Autism Dev Disord.

[10] Yirmiya N, Charman T (2010). The prodrome of autism: early behavioral and biological signs, regression, peri- and post-natal development and genetics. J Child Psychol Psychiatry.

[11] Ozonoff S (2010). A prospective study of the emergence of early behavioral signs of autism. J Am Acad Child Adolesc Psychiatry.

[12] Turner-Brown LM, Baranek GT, Reznick JS, Watson LR, Crais ER (2013). The first year inventory: a longitudinal follow-up of 12-month-old to 3-year-old children. Autism.

[13] Chevallier C, Kohls G, Troiani V, Brodkin ES, Schultz RT (2012). The social motivation theory of autism. Trends Cogn Sci.

[14] Dawson G, Meltzoff AN, Osterling J, Rinaldi J, Brown E (1998). Children with autism fail to orient to naturally occurring social stimuli. J Autism Dev Disord.

[15] Dawson G, Webb SJ, McPartland J (2005). Understanding the nature of face processing impairment in autism: insights from behavioral and electrophysiological studies. Dev Neuropsychol.

[16] Mundy P & Neal RA. in International review of research in mental retardation (ed. Laraine Masters Glidden) Volume 23, 139–168 (Academic Press, 2000).

[17] Shic F, Macari S, Chawarska K (2014). Speech disturbs face scanning in 6-month-old infants who develop autism spectrum disorder. Biol Psychiatry.

[18] Chawarska K, Macari S, Shic F (2013). Decreased spontaneous attention to social scenes in 6-month-old infants later diagnosed with autism spectrum disorders. Biol Psychiatry.

[19] Elsabbagh M (2013). The development of face orienting mechanisms in infants at-risk for autism. Behav Brain Res.

[20] Elsabbagh M, et al. What you see is what you get: contextual modulation of face scanning in typical and atypical development. Soc. Cogn. Affect. Neurosci. nst012 (2013). doi:10.1093/scan/nst012 10.1093/scan/nst012 PMC3989131 23386743

[21] Jones W & Klin A. Attention to eyes is present but in decline in 2-6-month-old infants later diagnosed with autism. Nature 2013; 504.7480; 427–431. 10.1038/nature12715 PMC4035120 24196715

[22] Webb SJ (2010). Toddlers with elevated autism symptoms show slowed habituation to faces. Child Neuropsychol J Norm Abnorm Dev Child Adolesc.

[23] Shah P, Gaule A, Bird G, Cook R (2013). Robust orienting to protofacial stimuli in autism. Curr Biol.

[24] Chawarska K, Volkmar F, Klin A (2010). Limited attentional bias for faces in toddlers with autism spectrum disorders. Arch Gen Psychiatry.

[25] Bloom LC, Mudd SA (1991). Depth of processing approach to face recognition: a test of two theories. J Exp Psychol Learn Mem Cogn.

[26] Chawarska K, Klin A, Volkmar F (2003). Automatic attention cueing through eye movement in 2-year-old children with autism. Child Dev.

[27] Webb SJ (2011). Developmental change in the ERP responses to familiar faces in toddlers with autism spectrum disorders versus typical development. Child Dev.

[28] Colombo J, Cheatham CL (2006). The emergence and basis of endogenous attention in infancy and early childhood. Adv Child Dev Behav.

[29] Colombo J & Mitchell DW. in Individual differences in infancy: reliability, stability, prediction 193–227 (New York: Lawrence Erlbaum Associates Publishers, 1990).

[30] Wass SV (2014). Comparing methods for measuring peak look duration: are individual differences observed on screen-based tasks also found in more ecologically valid contexts?. Infant Behav Dev.

[31] Gilmore RO, Thomas H (2002). Examining individual differences in infants’ habituation patterns using objective quantitative techniques. Infant Behav Dev.

[32] Kaplan PS & Werner JS. in Newborn attention: biological constraints and the influence of experience 278–307. (New York: Ablex Publishing, 1991).

[33] Colombo J, Mitchell DW (2009). Infant visual habituation. Neurobiol Learn Mem.

[34] Kaplan PS, Goldstein MH, Huckeby ER, Cooper RP (1995). Habituation, sensitization, and infants’ responses to motherese speech. Dev Psychobiol.

[35] Kaplan PS, Jung PC, Ryther JS, Zarlengo-Strouse P (1996). Infant-directed versus adult-directed speech as signals for faces. Dev Psychol.

[36] Elsabbagh M (2012). Infant neural sensitivity to dynamic eye gaze is associated with later emerging autism. Curr Biol.

[37] de Haan M, Nelson CA (1997). Recognition of the mother’s face by six-month-old infants: a neurobehavioral study. Child Dev.

[38] Nelson CA (2000). Neurocognitive sequelae of infants of diabetic mothers. Behav Neurosci.

[39] Richards JE (2003). Attention affects the recognition of briefly presented visual stimuli in infants: an ERP study. Dev Sci.

[40] de Haan M, Johnson MH, Halit H (2003). Development of face-sensitive event-related potentials during infancy: a review. Int J Psychophysiol Off J Int Organ Psychophysiol.

[41] Dawson G (2002). Neural correlates of face and object recognition in young children with autism spectrum disorder, developmental delay, and typical development. Child Dev.

[42] Halit H, de Haan M, Johnson MH (2003). Cortical specialisation for face processing: face-sensitive event-related potential components in 3- and 12-month-old infants. Neuroimage.

[43] de Haan M, Pascalis O, Johnson MH (2002). Specialization of neural mechanisms underlying face recognition in human infants. J Cogn Neurosci.

[44] de Haan M, Nelson CA (1999). Brain activity differentiates face and object processing in 6-month-old infants. Dev Psychol.

[45] Libertus K, Sheperd KA, Ross SW, Landa RJ. Limited Fine Motor and Grasping Skills in 6-Month-Old Infants at High Risk for Autism. Child Dev. 2014;85:2218–2231. 10.1111/cdev.12262 PMC4236283 24978128

[46] Estes A et al. Behavioral, cognitive, and adaptive development in infants with autism spectrum disorder in the first 2 years of life. J Neurodev Disord. 2015;7:1–10. 10.1186/s11689-015-9117-6 PMC4511527 26203305

[47] Iverson JM, Wozniak RH (2007). Variation in vocal-motor development in infant siblings of children with autism. J Autism Dev Disord.

[48] Johnson MH, Jones EJH & Gliga T. Brain adaptation and alternative developmental trajectories. Dev. Psychopathol. (in press). 10.1017/S0954579415000073 25997763

[49] Rankin CH (2009). Habituation revisited: an updated and revised description of the behavioral characteristics of habituation. Neurobiol Learn Mem.

[50] Bornstein MH, Benasich AA (1986). Infant habituation: assessments of individual differences and short-term reliability at five months. Child Dev.

[51] Casasola M, Bhagwat J (2007). Do novel words facilitate 18-month-olds’ spatial categorization?. Child Dev.

[52] Lewkowicz DJ (2004). Perception of serial order in infants. Dev Sci.

[53] Saffran JR, Pollak SD, Seibel RL, Shkolnik A (2007). Dog is a dog is a dog: infant rule learning is not specific to language. Cognition.

[54] Cohen LB, Atkinson DJ & Chaput HH. Habit X: a new program for obtaining and organizing data in infant perception and cognition studies. 2004.

[55] McCall RB (1979). Individual differences in the pattern of habituation at 5 and 10 months of age. Dev Psychol.

[56] Colombo J, Mitchell DW, Horowitz FD (1988). Infant visual attention in the paired-comparison paradigm: test-retest and attention-performance relations. Child Dev.

[57] Colombo J, Mitchell DW, O’Brien M, Horowitz FD (1987). The stability of visual habituation during the first year of life. Child Dev.

[58] Rose SA, Feldman JF, Wallace IF (1988). Individual differences in infants’ information processing: reliability, stability, and prediction. Child Dev.

[59] Dawson G (2012). Early behavioral intervention is associated with normalized brain activity in young children with autism. J Am Acad Child Adolesc Psychiatry.

[60] Elsabbagh M (2009). Neural correlates of eye gaze processing in the infant broader autism phenotype. Biol Psychiatry.

[61] Webb SJ, Dawson G, Bernier R, Panagiotides H (2006). ERP evidence of atypical face processing in young children with autism. J Autism Dev Disord.

[62] Reynolds GD, Richards JE (2005). Familiarization, attention, and recognition memory in infancy: an event-related potential and cortical source localization study. Dev Psychol.

[63] Robledo M, Deak GO & Kolling T. in Proceedings of the 32nd Annual Conference of the Cognitive Science Society 2482–2487 (Austin, TX: Cognitive Science Society, 2010).

[64] McCleery JP, Akshoomoff N, Dobkins KR, Carver LJ (2009). Atypical faces vs. object processing and hemispheric asymmetries in 10-month-old infants at risk for autism. Biol Psychiatry.

[65] American Psychological Association. Diagnostic and statistical manual of mental disorders—text revision. (2000).

[66] Kim SH, Lord C (2012). New autism diagnostic interview-revised algorithms for toddlers and young preschoolers from 12 to 47 months of age. J Autism Dev Disord.

[67] Landa R, Garrett-Mayer E (2006). Development in infants with autism spectrum disorders: a prospective study. J Child Psychol Psychiatry.

[68] Macari SL (2012). Predicting developmental status from 12 to 24 months in infants at risk for autism spectrum disorder: a preliminary report. J Autism Dev Disord.

[69] Nadig AS (2007). A prospective study of response to name in infants at risk for autism. Arch Pediatr Adolesc Med.

[70] Ozonoff S (2008). Atypical object exploration at 12 months of age is associated with autism in a prospective sample. Autism Int J Res Pract.

[71] Shen MD, et al. Early brain enlargement and elevated extra-axial fluid in infants who develop autism spectrum disorder. Brain awt166 (2013). doi:10.1093/brain/awt166 10.1093/brain/awt166 PMC3754460 23838695

[72] Young GS, Merin N, Rogers SJ, Ozonoff S (2009). Gaze behavior and affect at 6 months: predicting clinical outcomes and language development in typically developing infants and infants at risk for autism. Dev Sci.

[73] Colombo J, Shaddy DJ, Richman WA, Maikranz JM, Blaga OM (2004). The developmental course of habituation in infancy and preschool outcome. Infancy.

[74] Karmiloff-Smith A (1998). Development itself is the key to understanding developmental disorders. Trends Cogn Sci.

[75] Reynolds GD, Zhang D, Guy MW (2013). Infant attention to dynamic audiovisual stimuli: look duration from 3 to 9 months of age. Infancy.

[76] Jones EJH, Pascalis O, Eacott MJ, Herbert JS (2011). Visual recognition memory across contexts. Dev Sci.

[77] Colombo J, et al. in Infant pathways to language 143–168 (Hove, UK: Psychology Press, 2009).

[78] Webb SJ, Jones EJH, Kelly J, Dawson G (2014). The motivation for very early intervention for infants at high risk for autism spectrum disorders. Int J Speech Lang Pathol.

[79] Wass SV. et al. Shorter spontaneous fixation durations in infants with later emerging autism. Nat. Sci. Rep. (2015). doi:10.1038/srep08284 10.1038/srep08284 PMC4319149 25655672

[80] Colombo J (2001). The development of visual attention in infancy. Annu Rev Psychol.

[81] Colombo J, Richman WA, Shaddy DJ, Greenhoot AF, Maikranz JM (2001). Heart rate-defined phases of attention, look duration, and infant performance in the paired-comparison paradigm. Child Dev.

[82] Johnson MH (2014). Autism: demise of the innate social orienting hypothesis. Curr Biol.

[83] Wass S (2011). Distortions and disconnections: disrupted brain connectivity in autism. Brain Cogn.

[84] Keehn B, Wagner JB, Tager-Flusberg H, Nelson CA (2013). Functional connectivity in the first year of life in infants at-risk for autism: a preliminary near-infrared spectroscopy study. Front Hum Neurosci.

[85] Orekhova EV (2014). EEG hyper-connectivity in high-risk infants is associated with later autism. J Neurodev Disord.

[86] Wolff JJ (2012). Differences in white matter fiber tract development present from 6 to 24 months in infants with autism. Am J Psychiatry.

[87] Daluwatte C (2013). Atypical pupillary light reflex and heart rate variability in children with autism spectrum disorder. J Autism Dev Disord.

[88] Nyström P, Gredebäck G, Bölte S, Falck-Ytter T, EASE team (2015). Hypersensitive pupillary light reflex in infants at risk for autism. Mol Autism.

[89] Del Rosario M, Gillespie-Lynch K, Johnson S, Sigman M, Hutman T (2014). Parent-reported temperament trajectories among infant siblings of children with autism. J Autism Dev Disord.

[90] Richards JE (2010). The development of attention to simple and complex visual stimuli in infants: behavioral and psychophysiological measures. Dev Rev DR.

[91] Horowitz FD, Paden L, Bhana K, Self P (1972). An infant-control procedure for studying infant visual fixations. Dev Psychol.

[92] Gliga T (2015). Enhanced visual search in infancy predicts emerging autism symptoms. Curr Biol.

[93] McPartland J, Dawson G, Webb SJ, Panagiotides H, Carver LJ (2004). Event-related brain potentials reveal anomalies in temporal processing of faces in autism spectrum disorder. J Child Psychol Psychiatry.

[94] Pascalis O (2005). Plasticity of face processing in infancy. Proc Natl Acad Sci U S A.

[95] Jones EJH, Venema K, Lowy R, Earl RK & Webb SJ. Developmental changes in infant brain activity during naturalistic social experiences: video versus live interactions. Dev. Psychobiol. (in press). 10.1002/dev.21336 PMC4615531 26219834

[96] Ruysschaert L, Warreyn P, Wiersema JR, Metin B, Roeyers H (2013). Neural mirroring during the observation of live and video actions in infants. Clin Neurophysiol Off J Int Fed Clin Neurophysiol.

[97] Barr R (2010). Transfer of learning between 2D and 3D sources during infancy: informing theory and practice. Dev Rev DR.

[98] Kuhl PK, Tsao F-M, Liu H-M (2003). Foreign-language experience in infancy: effects of short-term exposure and social interaction on phonetic learning. Proc Natl Acad Sci U S A.

[99] Elison JT (2013). White matter microstructure and atypical visual orienting in 7-month-olds at risk for autism. Am J Psychiatry.

[100] Barnes K, et al. One year old infants who develop autism symptoms show increased frontal brain activity while viewing nonsocial stimuli. Infancy (In press.).

[101] Dawson G (2010). Randomized, controlled trial of an intervention for toddlers with autism: the Early Start Denver Model. Pediatrics.

[102] Ozonoff S, et al. Diagnostic stability in young children at risk for autism spectrum disorder: a Baby Siblings Research Consortium study. J. Child Psychol. Psychiatry n/a–n/a (2015). doi:10.1111/jcpp.12421 10.1111/jcpp.12421 PMC4532646 25921776

[103] Cox A (1999). Autism spectrum disorders at 20 and 42 months of age: stability of clinical and ADI-R diagnosis. J Child Psychol Psychiatry.

[104] Stone WL (1999). Can autism be diagnosed accurately in children under 3 years?. J Child Psychol Psychiatry.

[105] Landa RJ, Stuart EA, Gross AL, Faherty A (2013). Developmental trajectories in children with and without autism spectrum disorders: the first 3 years. Child Dev.

