# Supplementary material for: Reduced engagement with social stimuli in 6-month-old infants with later autism spectrum disorder: a longitudinal prospective study of infants at high familial risk
Source: J Neurodev Disord. 2016 Mar 15;8:7. doi: 10.1186/s11689-016-9139-8 (PMC4791854; doi:10.1186/s11689-016-9139-8)

**Table S1: Descriptive and demographic information for participants included in the full sample.** Included are Mean (Standard Deviation) and *Range.* Statistical comparisons between ASD-Neg and HR-ASD+ are presented in the “Outcome” column.

Key: m months, vSS verbal standard scores, nvSS non-verbal standard scores.

|  | **LR Expt 1** | **LR-ASD-Neg** | **HR-ASD-Neg** | **HR-ASD+** | **ASD-Neg vs ASD+** |
| --- | --- | --- | --- | --- | --- |
| **N (female)** | 6m: 114 (51)  12m: 104 (50) | 22 (11) | 27 (5) | 12 (7) | p = 0.33 |
| **Age (m):**  6m  12m  24m | 6.4 (0.3)  12.6 (.4) | 6.2 (.47) *6-8*  11.9 (.53) *11-13*  *N/A* | 6.1 (0.27) *6-7*  12.1 (0.4) *11-13*  24.5 (1.1) *23-27* | 6.0 (0.0) *6-6*  12.2 (1.0) *11-15*  24.3 (0.7) *23-25* | n.s.  n.s.  n.s. |
| **MSEL vSS**  6m  12m  24m | 98.9 (1.4) *81-132*  97.5 (1.6) *74-121*  *N/A* | 97.6 (12.2) 80-118  99.3 (14.5) 78-127  N/A | 91.8 (13.0) *60-118*  93.4 (14.2) *73-127*  109.1 (17.2) *85-138* | 93.4 (5.0) *86-99*  81.8 (13.8) *59-108*  85.1 (20.3) *59-110* | n.s.  *p* = 0.003  *p* = 0.001 |
| **MSEL nvSS**  6m  12m  24m | 104.5 (1.3) 79-125  109.4 (2.1) 80-137  N/A | 106.9 (13.6) 76-128  122.7 (12.7) 91-146  N/A | 99.2 (14.0) *73-136*  117.9 (10.4) *96-136*  109.7 (12.2) *83-127* | 96.9 (7.03) *85-109*  104.0 (15.5) *81-132*  90.9 (13.4) *68-108* | n.s.  *p* < 0.001  *p* <0.001 |
| **ADOS 24m Social**  **Com**  **Total** | N/A  N/A  N/A | N/A  N/A  N/A | 1.9 (2.0*) 0-6*  1.1 (1.2) *0-5*  3.0 (2.4) *0-7* | 7.4 (3.0) *3-12*  3.3 (1.5) *1-6*  10.7 (4.1) *5-18* | *p* < 0.001  *p* < 0.001  *p* < 0.001 |

**Table S2: Summary of participant providing data for the habituation task.** Data are provided based on children who: (a) Missed the data collection visit (“missing data”); (b) Attended, participated in the habituation paradigm (“collected data”); and (c) Who met the habituation criterion (“valid data”) followed by invalid data in parentheses (“invalid”), which was reported by the experimenter as being not reliably collected or child behavior was not compliant with the testing procedure. (See main text ‘Habituation Procedure’ for further details of validity assessment). Of note, if the total of valid+invalid trials is fewer than the number of subjects with collected data, the difference represents infants who did not participate in that particular habituation task at that time-point (e,g., ran out of time in the session).

Key: ‘Avg #of valid per infant’ = Average number of valid habituations per infant; this is the average number of habituation sessions per infant that were included in data analysis (maximum = 4).

| **6 months** | **LR-Exp1 (n=51)** | **LR-ASD-Neg (n=22)** | **HR-ASD-Neg (n=27)** | **HR-ASD+ (n=12)** |
| --- | --- | --- | --- | --- |
| **Face Short Valid (Invalid)** | 48 (3) | 22 (0) | 21 (0) | 7 (0) |
| **Face Long Valid Data (Invalid)** | 49 (2) | 21 (0) | 22 (0) | 9 (1) |
| **>= 1 Face Valid Data** | 50 | 22 | 22 | 9 |
| **Toy Short Valid Data (Invalid)** | 49 (2) | 20 (1) | 17 (6) | 7 (2) |
| **Toy Long Valid Data (Invalid)** | 45 (6) | 20 (1) | 18 (4) | 8 (0) |
| **>= 1 Toy Valid Data** | 50 | 22 | 23 | 10 |
| **Avg #of valid per infant** | *M*=3.3 *(SD* = 1.1*)* | *M* = 3.8 (*SD*=.11) | *M*=2.9 (*SD*1.5) | *M*=2.7 (*SD*1.5) |
| **12 months** | **LR-Exp1 (n=55)** | **LR-ASD-Neg (n=22)** | **HR-ASD-Neg (n=27)** | **HR-ASD+ (n=12)** |
| **Face Short Valid (Invalid)** | 46 (9) | 22 (0) | 23 (3) | 12 (0) |
| **Face Long Valid Data (Invalid)** | 49 (6) | 20 (2) | 23 (0) | 11 (0) |
| **>= 1 Face Valid Data** | 51 | 22 | 26 | 12 |
| **Toy Short Valid Data (Invalid)** | 48 (7) | 22 (0) | 24 (2) | 8 (4) |
| **Toy Long Valid Data (Invalid)** | 48(7) | 22 (0) | 22 (3) | 12 (0) |
| **>= 1 Toy Valid Data** | 51 | 22 | 26 | 12 |
| **Avg #of valid per infant** | *M*=3.6 *(*SD =.78*)* | *M*=3.9 (*SD*=.1) | *M*=3.3 (*SD*1.2) | *M*=3.6 (*SD*.5) |

**Table S3: Summary of number of participants providing data for the event-related potential task.** Data are provided based on children who: (a) Missed the data collection visit (“missing data”); (b) Attended, participated in the ERP paradigm (“collected data”); (c) Failed to wear the net at the testing session (“fail to wear net”); (d) Who did not attend to enough trials during data collection (“poor visual attention”); or (e) Had significant artifact during attended trials (“data quality poor”). Means and standard deviations for number of trials attended (average number of trials visually fixated by all children who participated in the ERP procedure) and number of attended trials *with artifact free data* are presented.

| **6 months** | **LR-Exp1** | **LR-ASD-Neg (n=22)** | **HR-ASD-Neg (n=27)** | **HR-ASD+ (n=12)** |
| --- | --- | --- | --- | --- |
| **Subj w/ Missing Data** | 0 | 0 | 2 | 2 |
| **Subj w/ Collected Data** | 114 | 21 | 25 | 10 |
| **Subj Fail to wear net** | 0 | 0 | 0 | 0 |
| **Subj poor visual attention** | 17 | 3 | 2 | 2 |
| **Subj Data quality poor** | 46 | 6 | 8 | 2 |
| **Technical error** | 1 | 0 | 2 | 0 |
| **# with good ERP data** | 50 | 12 | 13 | 6 |
| **Mean (Standard Deviation)** | **Face Toy** | **Face Toy** | **Face Toy** | **Face Toy** |
| **# of pictures attended**  **(# viewed by all children tested)** | 34.3 34.9  (15.2) (15.6) | 36.7 35.1  (17.3) (16.9) | 32.7 33.4  (16.7) (16.1) | 24.6 25.6  (14.3) (16.2) |
| **#of valid trials in analysis**  **(# Data quality good, for children included in ERP analysis only)** | 17.9 18.3  (7.2) (7.0) | 19.9 19.5  (7.4) (11.2) | 16.8 16.7  (6.5) (5.7) | 17.3 17.7  (6.3) (6.3) |
| **12 months** | **LR-Exp1** | **LR-ASD-Neg** | **HR-ASD-Neg** | **HR-ASD+** |
| **Subj w/ Missing Data** | 0 | 0 | 1 | 0 |
| **Subj w/ Collected Data** | 101 | 22 | 26 | 11 |
| **Subj Fail to wear net** | 3 | 2 | 2 | 1 |
| **Subj poor visual attention** | 19 | 3 | 7 | 2 |
| **Subj Data quality poor** | 23 | 3 | 5 | 3 |
| **Technical error** | 0 | 0 | 0 | 0 |
| **# with good ERP data** | 59 | 14 | 12 | 5 |
| **Mean (Standard Deviation)** | **Face Toy** | **Face Toy** | **Face Toy** | **Face Toy** |
| **# of trials attended**  **(# viewed by all children tested)** | 33.7 36.2  (16.7) (15.0) | 32.9 32.5  (18.0) (18.3) | 26.8 25.6  (19.0) (17.9) | 25.4 24.0  (11.4) (12.6) |
| **#of valid trials in analysis**  **(# Data quality good, for children included in ERP analysis only)** | 20.6 20.2  (7.8) (7.1) | 17.7 16.8  (7.5) (6.0) | 21.6 19.8  (9.5) (7.9) | 21.0 22.2  (9.4) (11.5) |

.

**Table S4: Summary of habituation variables by Age, Risk Group, Outcome Group and Stimulus.** Figures are Mean (Standard Deviation). ICC = intraclass correlation coefficient for the look durations produced by the two coders during testing; Peak Look = duration of the longest look during the habituation period; Peak Position = average position of the longest look in the habituation function; Dishabituation = look duration to the novel stimulus minus duration of the last look during habituation, for short (1 second) and long (1 minute) delay tasks.

|  | **Face** |  |  |  | **Toy** |  |  |  |
| --- | --- | --- | --- | --- | --- | --- | --- | --- |
|  | **LR-Exp1** | **LR-ASD-Neg** | **HR-ASD-Neg** | **HR-ASD+** | **LR-Exp1** | **LR-ASD-Neg** | **HR-ASD-Neg** | **HR-ASD+** |
| **6 months** | | | | | | | | |
| **ICC** | .95 (.12) | .95 (.02) | .94 (0.11) | .88 (0.15) | .96 (.1) | .96 (.02) | .90 (0.14) | .89 (.21) |
| **Peak Look** | 22.6 (13.8) | 27.7 (4.5) | 28.8 (17.3) | 15.6 (5.7)* | 17.7 (13.0) | 17.4 (2.5) | 16.2 (7.4) | 12.5 (5.8)* |
| **Peak Position** | 2.7 (1.9) | 3.0 (.6) | 2.7 (1.7) | 4.6 (3.4)* | 2.8 (1.9) | 3.5 (.6) | 3.2 (2.4) | 2.1 (1.6) |
| **Dishabituation**  **(short delay)** | 5.0 (1.9) | -.14 (1.7) | 2.2 (4.5) | 4.5 (5.9) | 4.2 (7.3) | 4.9 (1.3) | 1.9 (4.4) | 1.9 (2.1) |
| **Dishabituation**  **(long delay)** | 4.5 (7.3) | 5.8 (1.8) | 2.6 (5.8) | 5.5 (6.2) | 5.0 (5.1) | 4.5 (1.1) | 4.3 (5.0) | 6.3 (7.1) |
| **12 months** | | | | | | | | |
| **ICC** | .93 (.22) | .96 (.01) | .98 (.04) | .97 (.03) | .96 (.1) | .9 (.02) | .97 (.05) | 1.0 (.00) |
| **Peak Look** | 13.9 (5.9) | 13.9 (.98) | 18.0 (10.6) | 15.8 (7.7) | 11.2 (5.3) | 13.5 (1.1) | 13.4 (5.4) | 16.3 (5.4) |
| **Peak Position** | 2.5 (2.2) | 2.5 (.3) | 2.3 (12) | 2.4 (1.2) | 2.3 (1.6) | 2.9 (.4) | 2.8 (2.4) | 2.5 (1.7) |
| **Dishabituation (short delay)** | 4.6 (6.2) | 5.4 (1.4) | 4.8 (6.3) | 5.2 (6.5) | 6.2 (7.1) | 7.6 (1.3) | 3.6 (4.3) | 5.4 (5.8) |
| **Dishabituation (long delay)** | 6.2 (7.0) | 4.5 (1.7) | 4.6 (3.2) | 10.6 (15.0) | 5.9 (5.4) | 6.9 (1.9) | 6.0 (7.1) | 6.5 (5.6) |

**Table S5: Summary of ERP variables by Age, Risk Group, Outcome Group, and Condition. Figures are Mean (Standard Deviation).** Key : Amp = Amplitude; Lat = Latency; L = Left; R = Right

| **6 months** | **Face** |  |  |  | **Toy** |  |  |  |
| --- | --- | --- | --- | --- | --- | --- | --- | --- |
|  | **LR-Exp1** | **LR-ASD-Neg** | **HR-ASD-Neg (n=13)** | **HR-ASD+**  **(n=6)** | **LR-Exp1** | **LR-ASD-Neg** | **HR-ASD-Neg (n=13)** | **HR-ASD+ (n=6)** |
| **P1 Amp L** | 7.8 (5.1) | 4.0 (5.4) | 7.1 (5.2) | 7.1 (6.1) | 5.7 (5.1) | 5.1 (3.8) | 8.1 (4.1) | 5.2 (2.9) |
| **P1 Amp R** | 9.1 (4.7) | 7.3 (3.9) | 8.0 (6.0) | 6.5 (6.7) | 7.4 (6.6) | 4.9 (3.8) | 6.4 (2.6) | 6.3 (26) |
| **N2 Amp L** | -1.8 (7) | -5.2 (5.6) | -2.9 (5.8) | -2.2 (6.6) | -2.9 (7.2) | -.5 (5.9) | -2.8 (7.8) | -1.8 (3.1) |
| **N2 Amp R** | -2.1 (6.9) | -.18 (3.6) | -1.9 (7.1) | -0.4 (6.8) | -0.8 (6.9) | -1.1 (3.7) | -2.3 (4.1) | 1.1 (4.5) |
| **P4 Amp L** | 15.1(7.7) | 14.6 (8.2) | 16.5 (6.7) | 12.2 (10.7) | 17.3 (9.8) | 19.5 (11.4) | 15.8 (9.4) | 20.9 (10.9) |
| **P4 Amp R** | 15.2 (8.6) | 15.3 (8.0) | 15.7 (7.5) | 18.2 (6.3) | 17.7 (8.4) | 12.1 (7.5) | 19.3 (6.1) | 19.9 (4.7) |
| **P1 Lat L** | 188.2 (44.8) | 194.8 (56.2) | 169.1 (52.4) | 168.9 (45.8) | 179.8 (49.9) | 179.0 (39.5) | 192.0 (44.1) | 159.3 (24.3) |
| **P1 Lat R** | 175.5 (39.5) | 163.3 (27.2) | 170.5 (33.0) | 144.6 (43.4) | 176.2 (36.2) | 175.9 (37.6) | 177.9 (29.5) | 150.4 (37.6) |
| **N2 Lat L** | 287.0 (47.0) | 241.8 (62.5) | 282.8 (42.3) | 247.9 (44.5) | 286.7 (43.0) | 248.1 (60.4) | 271.2 (46.1) | 237.6 (45.8) |
| **N2 Lat R** | 278.9 (40.8) | 254.6 (54.2) | 262.2 (30.0) | 247.5 (41.1) | 280.5 (43.8) | 260.9 (58.9) | 254.2 (48.2) | 233.9 (36.5) |
| **P4 Lat L** | 563.6 (130.3) | 573.1 (144.9) | 587.7 (142.6) | 413.9 (93.6)* | 569.5 (111.6) | 554.0 (115.8) | 575.1 (89.6) | 640.1 (132.1) |
| **P4 Lat R** | 529.6 (110.0) | 542.1 (114.1) | 585.5 (123.5) | 516.4 (113.9) | 552.9 (96.3) | 550.6 (158.2) | 560.1 (94.8) | 546.3 (53.4) |
| **12 months** | **Face** |  |  |  | **Toy** |  |  |  |
|  | **LR-Exp1** | **LR-ASD-Neg** | **HR-ASD-Neg (n=12)** | **HR-ASD+ (n=5)** | **LR-Exp1** | **LR-ASD-Neg** | **HR-ASD-Neg (n=12)** | **HR-ASD+ (n=5)** |
| **P1 Amp L** | 6.2 (4.0) | **6.9 (5.2)** | 5.9 (5.2) | 4.8 (2.3) | 7.8 (5.3) | 8.0 (6.1) | 8.1 (3.9) | 4 (2.4) |
| **P1 Amp R** | 8.3 (5.0) | 8.8 (5.1) | 6.4 (5.7) | 4.7 (2.2) | 7.7 (5.2) | 7.6 (5.4) | 6.7 (6.4) | 6.5 (5.1) |
| **N2 Amp L** | -2.7 (5.8) | -6.0 (8.9) | -1.4 (5.2) | -0.8 (5.7) | 1.6 (6.6) | -3.9 (6.4) | -1.0 (4.5) | -0.6 (4.8) |
| **N2 Amp R** | -1.2 (5.3) | -3.8 (4.9) | 0.0 (5.0) | -1.7 (4.6) | 0.3 (6.2) | -3.4 (8.5) | -0.1 (6.6) | 3.6 (3.7) |
| **P4 Amp L** | 14.7 (8.0) | 13.5 (9.3) | 13.8 (5.5) | 10.1 (4.2) | 20.4 (7.3) | 17.9 (8.0) | 17.0 (9.4) | 9.6 (2.1) |
| **P4 Amp R** | 14.9 (9.1) | 16.3 (8.3) | 13.7 (11.0) | 9.1 (4.4) | 17.7 (7.9) | 18.6 (10.8) | 17.7 (7.4) | 13 (4.9) |
| **P1 Lat L** | 158.9 (35.0) | 146.8 (28.3) | 156.6 (30.5) | 156.8 (72.9) | 174.8 (43.6) | 163.6 (56.3) | 158.6 (56.0) | 173.3 (55.3) |
| **P1 Lat R** | 162.3 (30.2) | 154.8(24.5) | 163.8 (36.3) | 114.0 (18.9) | 165.4 (43.5) | 154.6 (45.5) | 156.0 (32.9) | 171.2 (45.6) |
| **N2 Lat L** | 256.1 (47.0) | 268.3 (38.5) | 244.3 (50.6) | 257.3 (58.3) | 261.1 (45.9) | 249.7 (49.0) | 239.1 (63.4) | 264.9 (84.7) |
| **N2 Lat R** | 256.9 (37.6) | 260.2 (37.9) | 259.4 (66.4) | 231.7 (40.8) | 250.7 (53.6) | 255.5 (62.6) | 228.4 (37.7) | 205.3 (67.8) |
| **P4 Lat L** | 489.1 (78.1) | 455.0 (66.6) | 462.5 (76.5) | 492.4 (94.4) | 504.9 (70.3) | 507.9 (95.6) | 493.6 (76.5) | 541.1 (81.9) |
| **P4 Lat R** | 479.6 (76.3) | 486.9 (50.4) | 439.9 (86.5) | 480.2 (119.7) | 498.2 (81.9) | 545.9 (121.2) | 486.9 (74.4) | 469.9 (89.0) |

**Table S6: Summary of habituation and clinical variables by Cluster of infants.** Included are Mean (Standard Deviation) and *Range.* Statistical comparisons between ASD-Neg and HR-ASD+ are presented in the “Outcome” column.

Key: m months, Vine Soc SS = Vineland Socialization Standard Score; Vine. Com SS. = Vineland Communication Standard Score, Mullen vSS = Mullen verbal standard scores, Mullen nvSS = Mullen non-verbal standard scores. * indicates there are significant effects of cluster on that domain.

|  | **N (female)** | **Vine. Soc SS** | **Vine Com SS.** | **Mullen vSS** | **Mullen nvSS** | **ADOS Total** |
| --- | --- | --- | --- | --- | --- | --- |
| **Cluster 1 ASD+**    **ASD-** | 0  14 (3) [7 HR] | *N/A*  97.2 (8.2)  *84-111* | *N/A*  106.0 (9.4)  *91-121* | *N/A*  100.6 (15.1)  *89-133* | *N/A*  105.6 (10.5)  *93-123* | *N/A*  4.1 (3.0)  *0-9* |
| **Cluster 2 ASD+**    **ASD-** | 5 (2)  26 (11) [13 HR] | 100* (5.1)  *95-107*  99.5 (6.6)  *89-118* | 101.8 (6.6)  *94-110*  108.5 (7.2)  *97-125* | 90.8 (19.8)  *63-110*  109.5 (15.4)  *89-13* | 94.8 (10.6)  *82-108*  110.5 (12.3)  *83-127* | 10.0* (1.8)  *8-12*  3.2 (2.0)  *1-7* |
| **Cluster 3 ASD+**    **ASD-** | 4 (3)  2 (0) [0 HR] | 87.3* (3.9)  *82-91*  101 (1.4)  *100-102* | 92.5 (10.0)  *86-107*  111.0 (2.8)  *109-113* | 77.7 (25.7)  *59-107*  N/A | 83.7 (19.1)  *68-105*  N/A | 14.5* (3.1)  *12-19*  N/A |
| **Cluster 2 vs 3 for ASD+** |  | *F*(1,8) = 15.9, *p* = 0.007. | n.s. | n.s. | n.s. | F(1,8) = 6.23, p = 0.047. |
| **Cluster 1 vs 2 for ASD-Neg** |  | n.s. | n.s. | n.s. | n.s. | n.s. |

**Figure S1:** Location of electrodes used in the analysis for the P400 (left) and Nc (right) components.


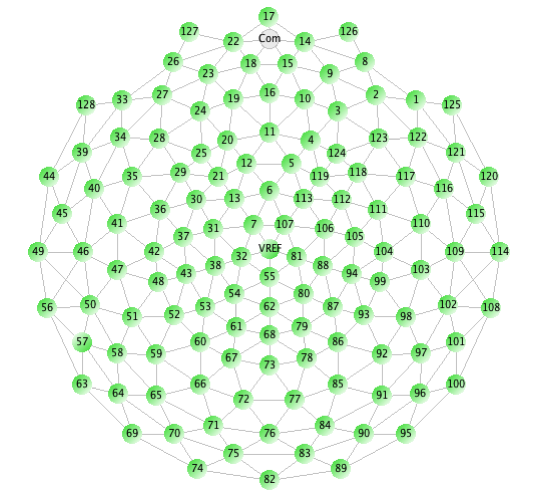

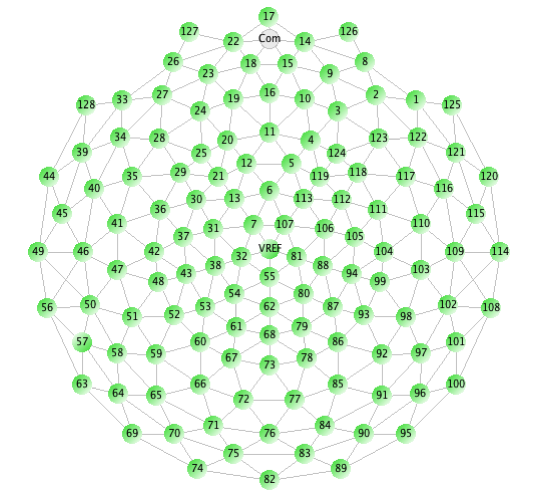


**Figure S2: Nc (A) and P400 (B) ERP components for the normative sample in Experiment 1.**

1. **Nc**

B) **400 Left and Right**

**
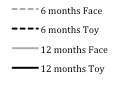
**

**Figure S3: Three dimensional scatter plot showing the three empirically identified clusters of infants.** Scores on the x axis are the peak look duration to objects at 6 months minus the peak look duration to objects at 12 months in seconds, with more positive scores representing a greater decrease in peak look time with age. Scores on the y axis are the peak look duration to faces at 6 months minus the peak look duration to faces at 12 months in seconds, with more positive scores representing a greater decrease in peak look time with age. Scores on the z axis are the position of the peak look in the sequence to faces minus the position of the peak look in the sequence to toys at 6 months. Positive scores represent a later peak look to faces.


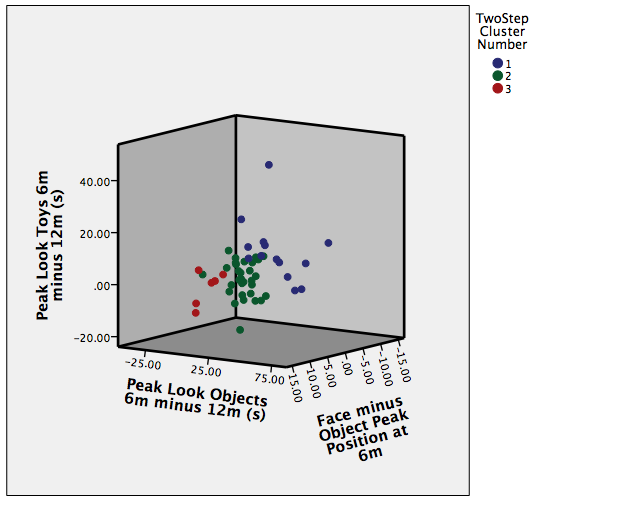

Supplement: Additional file 1: — Table S1. Descriptive and demographic information for participants included in the full sample. Table S2. Summary of participant providing data for the habituation task. Table S3. Summary of number of participants providing data for the event-related potential task. Table S4. Summary of habituation variables by Age, Risk Group, Outcome Group and Stimulus. Table S5. Summary of ERP variables by Age, Risk Group, Outcome Group, and Condition. Figures are Mean (Standard Deviation). Table S6. Summary of habituation and clinical variables by Cluster of infants. Figure S1. Location of electrodes used in the analysis for the P400 (left) and Nc (right) components. Figure S2. Nc (A) and P400 (B) ERP components for the normative sample in Experiment 1. Figure S3. Three dimensional scatter plot showing the three empirically identified clusters of infants. [file 11689_2016_9139_MOESM1_ESM.docx]
