# Supplementary material for: Reduced engagement with social stimuli in 6-month-old infants with later autism spectrum disorder: a longitudinal prospective study of infants at high familial risk
Source: J Neurodev Disord. 2016 Mar 15;8:7. doi: 10.1186/s11689-016-9139-8 (PMC4791854; doi:10.1186/s11689-016-9139-8)
Supplement: Additional file 2: — Supplementary text concerning methods and results. [file 11689_2016_9139_MOESM2_ESM.docx]

**1. Supplementary Materials Methods**

**S1.1. Participants**

Participants were recruited from a NIH-funded project examining the development of infants at high- or low- risk for autism. High Risk (HR) infants had an older sibling with ASD (n = 43; 15 female) and Low Risk (LR) infants had an older sibling without ASD or language impairment (n = 45; 19 female); infants participated in a range of tasks at 6, 12, 18, and 24 months. Demographic data is presented in Table S1.

Inclusion criteria for HR infant siblings included age (< 12 months), presence of autism in a full biological older sibling, and anticipated residence in the region (within 1.5 hours driving distance from the University) for the next 2 years. To confirm the diagnosis of ASD in an older sibling, the Autism Diagnostic Interview-Revised (ADI-R) was administered by phone and medical records were collected to confirm the diagnosis was based on DSM-IV criteria from a psychologist or physician.

Inclusion criteria for the LR group included age (< 12 months) and a biological older sibling without a diagnosis of ASD or language impairment; exclusionary criteria for non-risk infant siblings included a known family history of ASD in 1^st^ or 2^nd^ degree relatives. Additional exclusion criteria for the all participants included: physical signs (e.g., dysmorphic features) of known genetic syndromes, serious medical or neurological conditions (e.g., encephalitis, concussion, seizure disorder, diabetes, congenital heart disease), neurocutaneous markings, or sensory impairments such as vision or hearing loss; serious motor impairment; birth weight < 2000 grams and/or gestational age < 37 wks, history of intraventricular hemorrhage, exposure to neurotoxins (including alcohol, drugs), and maternal gestational diabetes. In addition, variables that may impact family functioning (e.g., serious parental substance abuse, bipolar disorder, or psychosis) were exclusion criteria.

**S1.2. Cognitive Assessment**

At each age, infants completed the Mullen Scales of Early Learning. The Mullen is a standardized assessment of early cognitive skills and was administered by a trained examiner. The Mullen is composed of five subscales assessing visual reception, gross and fine motor skills, and receptive and expressive language skills. Raw scores can be converted to t scores using standardization tables. We calculated standard scores for verbal (expressive language t-score + receptive language t-score) and nonverbal (fine motor t-score + visual reception t-score) skills. Group means, standard deviations and ranges are presented in Table S1.

**S1.3. ASD Assessment**

At 24 months, infants in the HR group were administered the ADOS module 1 (Lord et al., 2000). The ADOS is a play-based observation scale administered by a trained research reliable examiner. Infants are engaged in a semi-structured play with developmentally appropriate activities designed to elicit early social-communicative behaviors, language and communication, play and stereotyped/restricted behaviors or interests (see Table S1). Further, a clinical diagnosis was given as defined in the DSM-IV (American Psychiatric Association, 1994) by the consensus judgment of a certified clinical assessor and a licensed clinical psychologist, based on all available information obtained through the ADOS, cognitive testing, parental interview (the toddler adaptation of the ADI-R, Kim & Lord, 2012, plus any other information provided by the parent during testing) and any other experiences with the infants. Clinicians who were involved in diagnostic judgments are highly experienced in assessing ASD in toddlers of this age. Based on this information, infants were classified as having “Autistic Disorder”, “Pervasive Developmental Disorder- Not Otherwise Specified” or “no diagnosis”. Clinicians judged their confidence in the classification as “Very confident”, “Somewhat confident”, or “Not confident”. Of note, algorithm cut-offs on the ADI-R and ADOS were not used to determine DSM-IV judgments at this stage.

For analysis, infants within the HR group were further divided based on their diagnostic outcome at 24 months. Of the original group of 43 HR infants, 3 did not receive a 24-month assessment and were not included in analyses. Infants in the HR-ASD+ (n=12) group all met DSM IV criteria for ASD at 24 months. Where clinicians had judged that they were “Not confident” in this judgment, infants were additionally required to meet cut-off on the ADOS for ASD (n=1). Infants in the HR-ASD-Neg group were judged to have “no diagnosis” on DSM-IV criteria (n=27). One of these infants met cut-off for ASD on the ADOS (total score = 8), but was retained in the HR-ASD-Neg group because consensus clinical judgment indicated he did not meet DSM-IV criteria. One additional infant met DSM-IV criteria for ASD at 24 months but clinicians judged that they were “not confident”. This infant did not meet cut-off on the ADOS (total score = 5). He was also judged to “technically meet criteria, due to the presence of subtle delays”. Given the young age of the children and the need for confidence in ASD judgments, we chose to exclude this infant from analyses. Of note, inclusion of this child in the HR-ASD-Neg group did not alter the significance of results.

<<Insert Table S1 about here>>

**S1.4. Habituation Task**

*S1.4.1. Participants:* Table S2 presents the number of children from each group who provided valid data within each condition and age point. Presenting such information is important because any differential drop-out between groups could affect interpretation of data. Data are provided based on children who: (1) missed the data collection visit (“missing data”); (2) attended, participated in the habituation paradigm (“collected data”); and (3) who met the habituation criterion (“valid data”) followed by invalid data in parentheses (“invalid”), which was reported by the experimenter as being not reliably collected or child behavior was not compliant with the testing procedure.

For Experiment 2, at 6 and 12 months, the average number of valid habituations did not differ by ASD Outcome Group (*p*>0.5).

*S1.4.2.* *Habituation analysis strategy*: Table S4 gives descriptive statistics for all key habituation variables.

**S1.5. Event Related Potential (ERP) Task**

S1.5.1. *Participants*: Table S3 presents the number of children from each group who provided valid data for each age point. Data are provided based on children who: (1) missed the data collection visit (“missing data”); (2) attended, participated in the ERP paradigm (“collected data”); (3) failed to wear the net at the testing session (“fail to wear net”); (4) who did not attend to enough trials during data collection (“poor visual attention”); (5) had significant artifact during attended trials (“data quality poor”). Means and standard deviations for number of trials attended (average number of trials visually fixated by all children who participated in the ERP procedure ) and number of attended trials *with artifact free data* are presented. These values can be used to identify whether there are likely to be differences in the signal to noise ratio of ERP data between groups. Of note, these values are almost identical to other studies in this area (e.g. Elsabbagh et al., 2012).

There were no significant Outcome group differences in attended trials at 6 or 12 months (Fs < 2.7, ps > .1; see Supplemental Materials Table S3). Importantly, there were no significant differences in the final number of *attended,* *artifact-free trials* included in analysis between Outcome Groups (Fs < 0.1, ps > .8), or Stimulus categories at either age (Fs < 0.5, ps > .4; see Table S3).

**2. Supplementary Materials Results**

**S 2.1. Habituation: LR-ASD-Neg vs HR-ASD-Neg**

We examined whether there were any differences between infants without later ASD from the two risk groups (6m: HR-ASD-Neg n=21 and LR-ASD-Neg n=22; 12m HR-ASD-Neg n=25 and ASD-Neg n=22), to assess whether combining the LR-ASD-Neg and HR-ASD-Neg groups was appropriate. Descriptive data can be found in Supplementary Materials S2.1. and Supplementary Table S4. At both 6 and 12 months, there were no significant differences between risk groups in either peak look duration (Effect of Group 6m: F(1,39) = 0.08, p = 0.79; 12m: F(1,43) = 0.10, p = 0.8) or peak look position (6m: Stimulus by Group F(1,41) = 0.32, p = 0.86; Group F(1,39) = 1.0, p = 0.32; 12m: Stimulus by Group F(1,43) = 1.3, p = 0.27; Group F(1,43) = 0.006, p = 0.9).

**S2.2. Event-related potentials: LR-ASD-Neg vs HR-ASD-Neg**

We next examined whether there were any differences between infants without later ASD from the two risk groups (6m: HR-ASD-Neg n=13 and LR-ASD-Neg n=12; 12m: HR-ASD-Neg n=25 and ASD-Neg n=22), to identify possible ‘broader phenotype’ effects of familial risk status. At 6 and 12 months, there were no significant effects including Risk group (LR-Neg, HR-Neg) on P400 latency or amplitude, or Nc amplitude or duration (*F*s < 2, *p*s > 0.2).

**S2.3 Children included in ERP analyses at 6 versus 12m**

We used ANOVAs with developmental scores (Mullen, ADOS) as the dependent variables, and diagnostic group and inclusion group (No data, 6m data only, 12m data only or 6 and 12m data) as independent variables.

These analyses showed (as expected) that children with ASD had lower Mullen composite scores than children with no ASD at 12 (*F*(1,53) = 20.6, p < 0.001) and 24 months (*F*(1,29) = 23.0, p < 0.001), but not at 6 months (*F*(1,49) = 0.8, p =0.4). Also as anticipated, ADOS total scores were higher in children with ASD than children with no ASD (*F*(1,30) = 68.6, *p* < 0.001). Mullen composite scores at 6 months and 12 months did not vary by inclusion group (6m: *F*(3,49) = 0.09, *p* = 1.0; 12m: *F*(3,53) = 0.55, *p* = 0.65) and there was no significant interaction with Outcome group (6m: *F*(3,49) = 0.26, *p* = 0.9; 12m: *F*(3,53) = 1.5, *p* = 0.22). However, 24m Mullen composite scores did vary by Inclusion group (F(3,29) = 3.26, p = 0.04) but this did not interact significantly with Outcome Group (F(3,29) = 2.1, p = 0.12). Posthoc tests indicate that the group with 6m data only, and the group with 6 and 12m data did show higher Mullen scores at 24 months than the group with 12m data only (M 6m and 12m = 95.9; M 6m only = 107.8; M 12m only = 86.6; p = 0.05; p = 0.06). Similarly, 24m ADOS total scores varied by Longitudinal Inclusion group (F(3,30) = 3.7, p = 0.02) and this did significantly interact with Outcome Group (F(3,30) = 3.3, p = 0.03). Posthoc tests indicated that infants with data at 12m only significantly differed from infants with data at 6m only (M 12m only = 8.5; M 6m only = 5.3; p = 0.04) and those with no data at either timepoint (M = 4.8, p = 0.008). These differences were most extreme within the ASD group. Thus, infants with data at 12m only showed lower Mullen scores and higher ADOS scores at 24 months than infants with 6m data only, or infants with longitudinal data. If anything, this would be expected to magnify differences at 12m (since infants at that age were most impaired, and this was particularly the case for the ASD group), which does not match our pattern of findings.

**S2.4 Group effects on the P1 and N290 ERP components.**

We analyzed peak amplitude and latency for both the P1 and N290 components. As for the P400, peaks were identified for each electrode using automatic peak detection software, and verified by visual inspection. Peaks were defined as the most positive points of a deflection between 80 and 300ms (P1) and 150 and 400ms (N290), and the peak had to be present in at least 2/6 electrodes in a group. This time window was based on previous work and inspection of individual averages for the typically developing normative sample included in Experiment 1. Peak amplitude and latency values were averaged across regions.

1. **Experiment 2: ASD vs ASD-Neg.**

P1: There were no significant effects or interactions with group on P1 amplitude or latency (Fs < 1, ps > .4)

N1: There were no significant effects or interactions with group on N1 amplitude or latency (Fs < 1, ps > .4)

Thus, effects reported in the main text for P400 latency do not reflect cascading effects from previous components. Further, there is no evidence in the present dataset for differences in early perceptual processing in infants with later ASD.
